# Interplay of pathogenic forms of human tau with different autophagic pathways

**DOI:** 10.1111/acel.12692

**Published:** 2017-10-12

**Authors:** Benjamin Caballero, Yipeng Wang, Antonio Diaz, Inmaculada Tasset, Yves Robert Juste, Barbara Stiller, Eva‐Maria Mandelkow, Eckhard Mandelkow, Ana Maria Cuervo

**Affiliations:** ^1^ Department of Developmental and Molecular Biology Albert Einstein College of Medicine Bronx NY 10461 USA; ^2^ Institute for Aging Studies Albert Einstein College of Medicine Bronx NY 10461 USA; ^3^ German Center for Neurodegenerative Diseases (DZNE) 53175 Bonn Germany; ^4^ CAESAR Research Center 53175 Bonn Germany

**Keywords:** aging, autophagy, Alzheimer's disease, frontotemporal dementia, lysosomes, neurodegeneration

## Abstract

Loss of neuronal proteostasis, a common feature of the aging brain, is accelerated in neurodegenerative disorders, including different types of tauopathies. Aberrant turnover of tau, a microtubule‐stabilizing protein, contributes to its accumulation and subsequent toxicity in tauopathy patients’ brains. A direct toxic effect of pathogenic forms of tau on the proteolytic systems that normally contribute to their turnover has been proposed. In this study, we analyzed the contribution of three different types of autophagy, macroautophagy, chaperone‐mediated autophagy, and endosomal microautophagy to the degradation of tau protein variants and tau mutations associated with this age‐related disease. We have found that the pathogenic P301L mutation inhibits degradation of tau by any of the three autophagic pathways, whereas the risk‐associated tau mutation A152T reroutes tau for degradation through a different autophagy pathway. We also found defective autophagic degradation of tau when using mutations that mimic common posttranslational modifications in tau or known to promote its aggregation. Interestingly, although most mutations markedly reduced degradation of tau through autophagy, the step of this process preferentially affected varies depending on the type of tau mutation. Overall, our studies unveil a complex interplay between the multiple modifications of tau and selective forms of autophagy that may determine its physiological degradation and its faulty clearance in the disease context.

**Abbreviations**
AD: Alzheimer's disease
CMA: chaperone‐mediated autophagy
e‐MI: endosomal microautophagy
FTD: frontotemporal dementia
GFP: green fluorescent protein
hsc: heat‐shock cognate protein
LAMP: lysosome‐associated membrane protein
LC3: light chain 3 protein
LE: late endosomes
MVB: multivesicular bodies
N2a: Neuro‐2a neuroblastoma cell line
PHF: pair helical filament
PTM: posttranslational modifications
WT: wild‐type

## Introduction

It is well accepted that loss of proteostasis occurs gradually with age and underlies the basis of severe neurodegenerative disorders such as Parkinson's disease, Huntington's disease, Alzheimer's disease, and other types of frontotemporal dementia (Prahlad & Morimoto, 2009; Voisine *et al*., 2010; Morimoto & Cuervo, 2014). Aberrant proteins are normally handled by the proteostasis networks responsible for maintaining protein quality control (Kaushik & Cuervo, 2015). Among the proteolytic systems of these networks, autophagy or protein breakdown in lysosomes has proven effective in maintenance of a healthy proteome (Nixon, 2013). However, numerous evidences suggest that protein variants shown to contribute to the pathogenesis of neurodegenerative disorders may fail to undergo degradation by autophagy and accumulate intracellularly resulting in cell toxicity and often cell death (Nixon, 2013; Menzies *et al*., 2015). Defective autophagy is sometimes a consequence of modifications on the pathogenic proteins that prevent their autophagic removal, whereas in other instances, pathogenic proteins directly impair the autophagic systems (Wong & Cuervo, 2010).

Pathogenic variants of the protein tau induce a group of neurodegenerative conditions generically known as tauopathies. Although tauopathies are caused by mutations in the same protein, tau, different types of tauopathies appear to affect different brain regions and exhibit distinct symptoms, suggesting that tau mutations may have distinctive consequences. For instance, we recently revealed that tau mutations ΔK280 and A152T influence neuronal activity in an opposite manner (Decker *et al*., 2016; Dennissen *et al*., 2016). We wondered whether different tau mutations may also impact tau clearance differently. Our groups and others have shown the degradation of wild‐type forms of tau through the ubiquitin/proteasome system and through autophagy and failure to degrade some mutant forms of tau through these pathways (Lee *et al*., 2013; Holtzman *et al*., 2016; Wang & Mandelkow, 2016). However, the degradation pathways of different physiological tau isoforms and the effect of disease‐related point mutations and posttranslational modifications on tau degradation remain largely unknown. We have previously found that chaperone‐mediated autophagy (CMA)—a selective form of autophagy in which substrate proteins directly cross the lysosomal membrane for degradation and known to decrease with age—contributes under physiological conditions to efficient turnover of unmodified tau protein but that mutations in tau prevent its degradation by CMA (Wang *et al*., 2009). These findings made us consider that other modifications of tau, including mutations associated with disease, could also impact the degradation pathways involved in their degradation and accelerate the underlying age‐dependent decrease in autophagic activity.

In this work, we have systematically measured the degradation of different forms of tau by each of the three main types of autophagy described in mammals, macroautophagy, chaperone‐mediated autophagy (CMA), and endosomal microautophagy (e‐MI); and analyzed the interaction of tau proteins with the vesicular compartments that participate in each autophagic pathway and their impact on these pathways. We first compared two different mutations: P301L, known to cause autosomal‐dominant frontotemporal dementia (FTD) (Hutton *et al*., 1998); and a point mutation of tau (A152T) that does not cause autosomal‐dominant disease but associates with higher risk of frontotemporal dementia and Alzheimer's disease (AD) (Coppola *et al*., 2012). Our work reveals that both mutant forms of tau become poor e‐MI substrates and that P301L mutation, in addition, markedly reduces tau's susceptibility for CMA degradation. Cells respond to expression of these mutants by upregulating macroautophagy, and to a lesser extent CMA, but this upregulation is no longer sustained when cells are challenged with additional stressors, suggesting that there is an inability of cells to handle additional stress in the presence of these tau mutants.

In the second part of this study, we compared tau isoforms with different numbers of repeats (3R vs 4R) or with previously characterized mutations known to affect different tau properties to analyze the possible impact of these changes in the relation of tau with the different autophagic pathways. We included the following: (i) deletion of lysine 280 (ΔK280) (Rizzu *et al*., 1999; Momeni *et al*., 2009), which leads to excess of 3R transcripts (van Swieten *et al*., 2007) and enhances tau aggregation into PHFs (Rizzu *et al*., 1999; Barghorn *et al*., 2000); (ii) mutations in cysteines 291 and 322 (C291 and C322), whose oxidation increases propensity of tau aggregation (Barghorn & Mandelkow, 2002; Mo *et al*., 2009); and (iii) mutations that mimic or disrupt phosphorylation in residues previously related to tau toxicity or aggregation propensity (Biernat & Mandelkow, 1999). We have found that these modifications have a different impact on the degradation of tau by each of the autophagic pathways, and on the way in which they affect functioning of these autophagic pathways. Overall, our studies unveil a complex interplay between the multiple modifications of tau and selective forms of autophagy. This suggests that some of these modifications play regulatory roles in the physiological clearance of tau through these pathways, whereas others may compromise normal routes of autophagic clearance of tau and thus contribute to pathogenesis.

## Results

### Altered CMA of tau‐P301L

**Figure 1:**
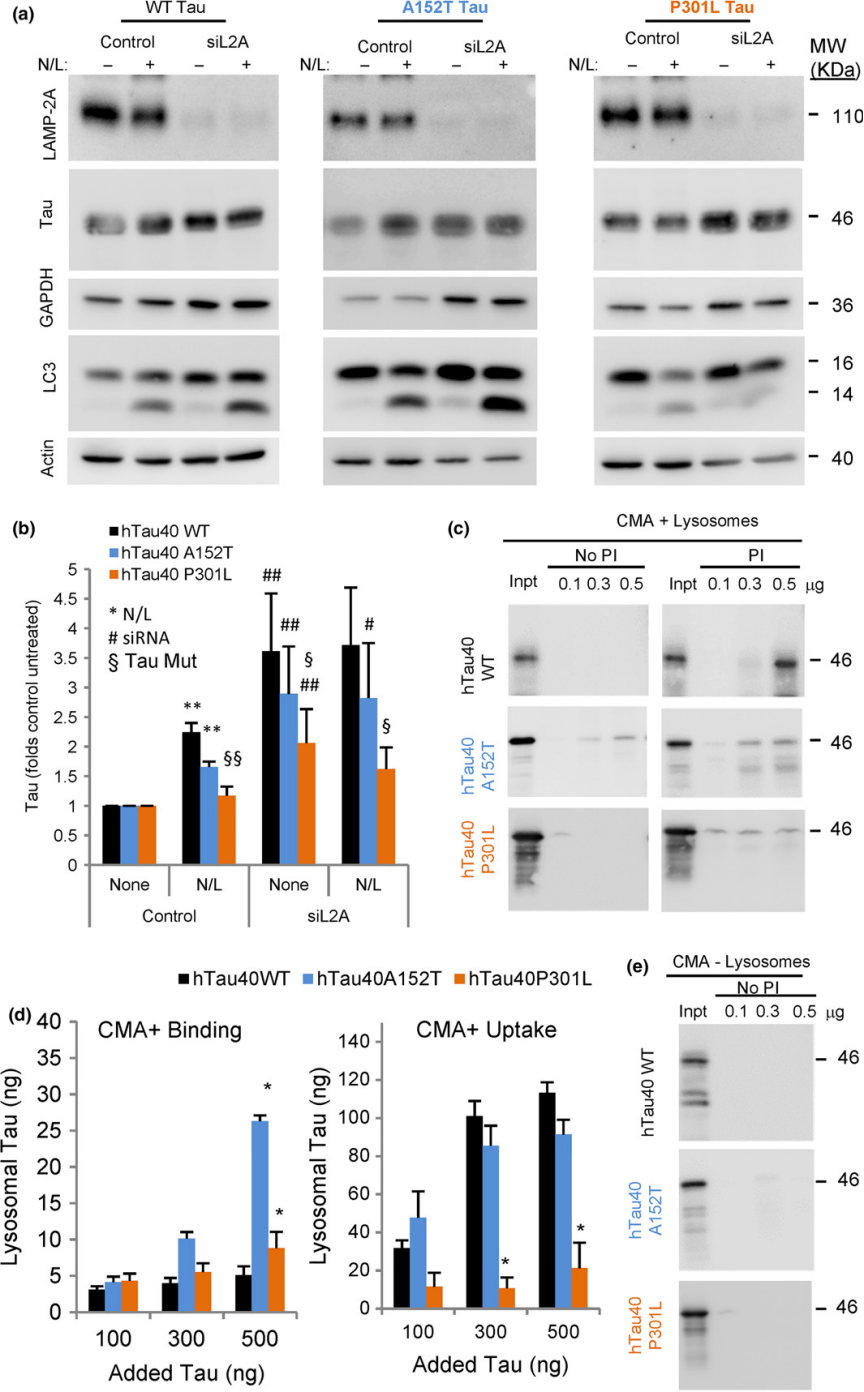
Contribution of CMA to degradation of disease‐related mutant tau proteins. (a) Immunoblots for the indicated proteins of the mouse neuroblastoma cell line Neuro‐2a (N2a) control or knockdown for LAMP2A (siL2A) expressing under the control of a tetracycline promoter tau wild‐type (WT) or tau mutated at residues A152T or P301L. Cells were treated with doxycycline to activate protein expression for 72 h and, where indicated, NH_4_Cl 20 mm and leupeptin 100 μm (N/L) were added during the last 4 h of incubation. LC3‐II levels are shown as positive control of the effect of the inhibitors. GAPDH is shown as an example of well‐characterized CMA substrate. Actin is shown for normalization purposes (note that lower relative contribution of actin in the same amount of total protein loaded is a consequence of the accumulation of proteins no longer degraded when CMA is blocked). (b) Quantification of tau levels normalized to actin. Values are expressed relative to those in untreated control cells that were given an arbitrary value of 1. *n* = 5. Differences after adding N/L (*), upon siRNA (#) or of the mutant tau proteins relative to WT (§) were significant for *,#,§*P* < 0.05 and **,##,§§*P* < 0.01. (c) Immunoblots for tau of isolated CMA‐active lysosomes, pretreated or not with protease inhibitors (PI) for 10 min at 4 °C and then incubated with the indicated tau proteins at 37 °C for 20 min. Inpt: input (0.1 μg). (d) Quantification of binding (left) and uptake (right) of tau proteins by the CMA‐active lysosomes. Values are indicated in ng, and were calculated from the densitometric quantification of a known amount of purified protein. *n* = 5. (e) Immunoblot for tau proteins incubated under the same condition as in c but with CMA‐inactive (−) lysosomes. Input = 0.1 μg. All values are mean ± SEM. Differences with hTau40 WT were significant for *P < 0.05.

An increase in overall tau levels has been observed in brains from patients bearing either P301L or A152T mutation on tau (Torres *et al*., 1998). However, whereas the P301L mutation leads to tau aggregation into paired helical filaments (PHFs) (Barghorn *et al*., 2000), patients with the risk‐associated A152T mutation display higher abundance of oligomers (Coppola *et al*., 2012). To analyze whether the differences in the behavior and cellular toxicity of these two tau mutants could be in part attributable to mutation‐specific changes in tau degradation, we first measured the contribution of CMA to their clearance. We used the mouse neuroblastoma cell line, Neuro‐2a (N2a), stably expressing under the control of a tetracycline‐regulated promoter full‐length (441 residues) human tau (hTau40, which indicates tau protein with 2N and 4R domains (2N4R‐tau)) as wild‐type (WT) or with mutations A152T or P301L (Khlistunova *et al*., 2006). The CMA receptor LAMP‐2A was knocked down using siRNA (Massey *et al*., 2006). In agreement with our previous observations (Wang *et al*., 2009), we found that lysosomes contribute to degradation of WT tau (reflected as an increase in tau levels upon blockage of lysosomal proteolysis with inhibitors). This lysosomal degradation occurred, in large part, through CMA, as genetic blockage of this pathway almost completely abolished lysosomal degradation of WT tau and led to its accumulation (Fig. 1a,b; GAPDH is shown as an example of a well‐characterized CMA substrate (Aniento *et al*., 1993) known to accumulate intracellularly upon blockage of CMA (Schneider *et al*., 2014)). Tau‐A152T displayed very similar degradation dynamics, although this mutation slightly reduced tau's rates of lysosomal degradation (20% inhibition) when compared with WT tau (Fig. 1a,b). Blockage of CMA in cells expressing tau‐A152T also resulted in significant accumulation of this variant and ablated its lysosomal degradation, suggesting preferential degradation of A152T by CMA (Fig. 1a,b). In contrast, tau‐P301L did not significantly accumulate upon blockage of lysosomal proteolysis, indicative of minor contribution of the lysosomal system to the clearance of this mutant form of tau (Fig. 1a,b). Interestingly, although tau‐P301L was not degraded in lysosomes, blockage of CMA promoted accumulation of this protein variant, albeit at significantly lower levels than WT and A152T. We propose that overall loss of proteostasis as a consequence of CMA blockage could indirectly affect clearance of tau‐P301L through other systems.

To further elucidate the contribution of CMA to the degradation of the two mutants in the absence of any other proteolytic system, we used a well‐established *in vitro* system that allows to recapitulate different CMA steps (binding and translocation of substrates) using isolated intact lysosomes (Kaushik & Cuervo, 2009). We presented lysosomes with either purified WT, A152T, or P301L tau and incubated them in the presence or absence of protease inhibitors to block tau degradation (Fig. 1c,d). This allows determining lysosomal binding of tau as the amount of tau at the end of the incubation associated with the group of lysosomes not pretreated with protease inhibitors, as internalized tau would be rapidly degraded. Uptake of tau was calculated by the difference between the amount of tau associated with lysosomes pretreated with protease inhibitors and those not pretreated. Consistent with our previous findings (Wang *et al*., 2009), WT tau is a very efficient CMA substrate, to the point that binding is almost undetectable because the protein is rapidly internalized (Fig. 1c,d). In contrast, the P301L mutation severely impaired lysosomal uptake of tau by CMA, resulting in a sixfold decrease in degradation when compared to WT tau protein (Fig. 1c,d). Reduced tau‐P301L uptake is not caused by a problem in translocation across the lysosomal membrane, but rather by reduced targeting/binding to lysosomes, as we did not detect tau accumulation at the lysosomal membrane. This is in clear contrast to other pathogenic proteins such as mutant α‐synuclein or mutant LRRK2 that bind to lysosomes but fail to translocate through CMA (Cuervo *et al*., 2004; Orenstein *et al*., 2013). In the case of tau‐A152T, the dynamics of internalization/degradation through CMA were comparable to WT tau (Fig. 1c,d), in agreement with our studies in intact cells in culture (Fig. 1a,b), but we found a significantly higher amount of tau‐A152T bound to the membrane of CMA‐active lysosomes (Fig. 1c,d). To determine whether this abnormally enhanced binding was due to nonselective association of the tau mutant with membranes in general, we incubated the three proteins with lysosomes inactive for CMA (they have a very similar membrane composition to the CMA‐active pool of lysosomes, but lack hsc70 in their lumen). As shown in Fig. 1e, higher binding of tau‐A152T was no longer observed when using CMA‐incompetent lysosomes, thus supporting that the enhanced binding was related to CMA uptake. We propose that the higher amount of tau‐A152T bound to the lysosomal membrane at a given time may be a consequence of its slightly reduced translocation rate into the lumen when compared to WT tau (Fig. 1c,d).

Taken together, our *in vitro* and cell‐based studies argue that these two point mutations, A152T and P301L, reduce the normal degradation of tau by CMA, although the P301L mutation has a more pronounced inhibitory effect.

### Reduced e‐MI of pathogenic tau point mutants

**Figure 2:**
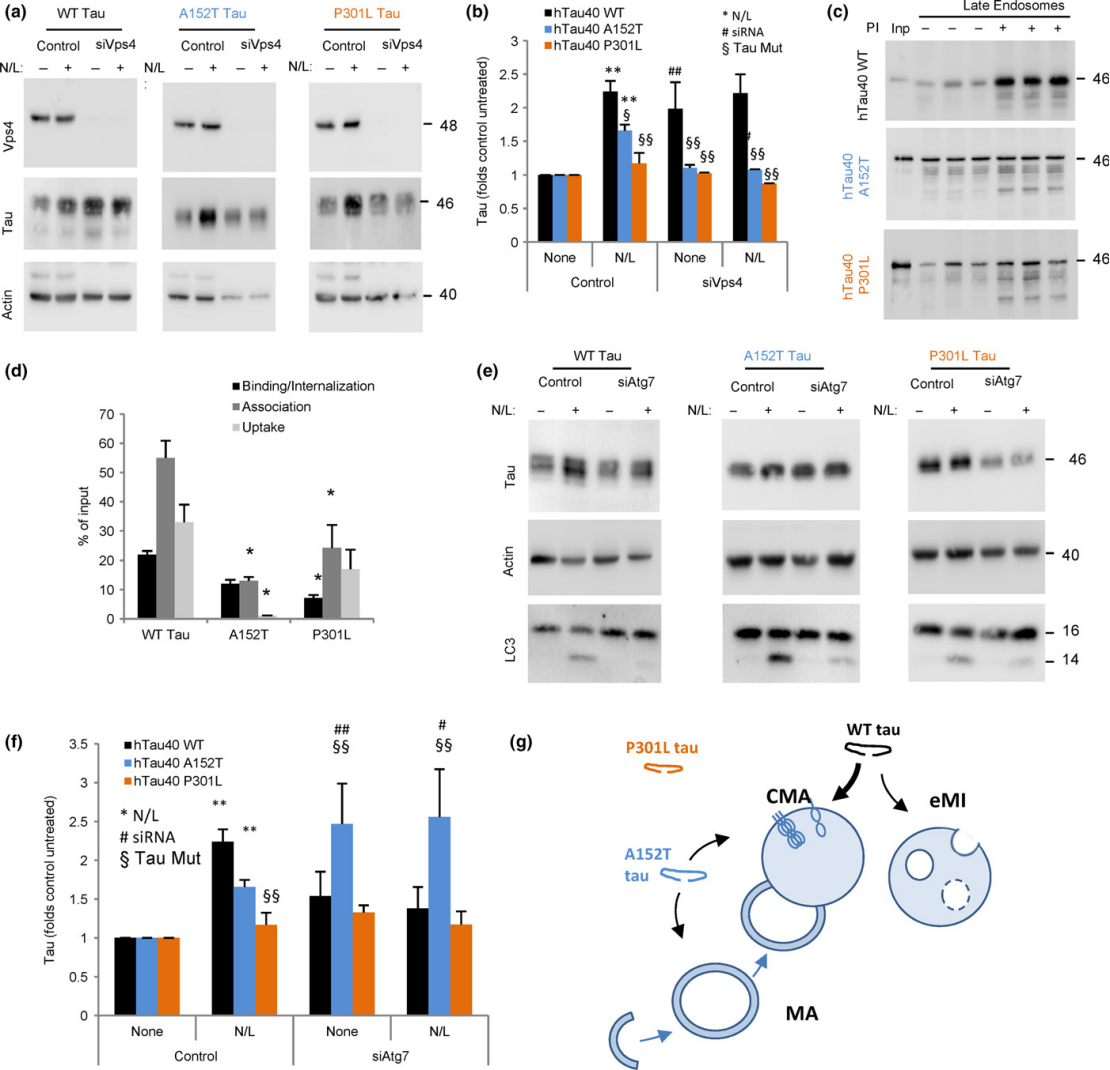
Endosomal microautophagy and macroautophagy of disease‐related mutant tau proteins. (a) Immunoblots for the indicated proteins of the mouse neuroblastoma cell line Neuro‐2a (N2a) control or knockdown for Vps4 A and B (siVps4) expressing under the control of a tetracycline promoter tau wild‐type (WT) or tau mutated at residues A152T or P301L Cells were treated with doxycycline to activate protein expression for 72 h and, where indicated, NH_4_Cl 20 mm and leupeptin 100 μm (N/L) were added during the last 4 h of incubation. Actin is shown for normalization purposes (lower relative contribution of actin to total cellular protein in the knockdown cells is a consequence of the accumulation of proteins no longer degraded when e‐MI is blocked in the mutant‐expressing cells). (b) Quantification of tau levels relative to those in untreated control cells that were given an arbitrary value of 1. *n* = 4. Differences after adding N/L (*), upon siRNA (#) or of the mutant tau proteins relative to WT (§) were significant for *,#,§*P* < 0.05 and **,##,§§*P* < 0.01. (c) Immunoblots for tau associated with isolated late endosomes pretreated or not with protease inhibitors (PI) for 10 min at 4 °C and then incubated with the indicated tau proteins at 37 °C for 30 min. Inp: input. (d) Quantification of binding, association, and uptake/degradation of tau proteins by the late endosomes. Values are indicated as percentage of the input, calculated from the densitometric quantification of a known amount of purified protein. *n* = 4. Differences with hTau40 WT were significant for *P < 0.05. (e) Immunoblots for the indicated proteins of the same cells as in (a) but knockdown for Atg7 (siAtg7). (f) Quantification of tau levels as in (b). *n* = 3. Significance of the differences is expressed as in (b). All values are mean ± SEM. (g) Scheme of the contribution of different autophagic pathways to the degradation of WT, P301L or A152T mutant tau.

Late endosomes (LE) also contribute to selective degradation of cytosolic proteins targeted to this compartment by hsc70 through a process known as endosomal microautophagy (e‐MI) (Sahu *et al*., 2011; Uytterhoeven *et al*., 2015). Using the same N2a cells expressing WT, A152T, and P301L tau, we analyzed changes in cellular levels of these forms of tau upon disrupting e‐MI. To that effect, we knocked down essential components of the endosomal sorting complex required for transport (ESCRT) (Sahu *et al*., 2011). Contrary to WT tau, which accumulates in e‐MI‐defective cells, intracellular levels of A152T and P301L tau did not change in cells knocked down for Vps4, suggesting that both point mutations in tau compromise its ability to undergo degradation by this pathway (Fig. 2a,b). Unexpectedly, although A152T and P301L did not accumulate in Vps4 knockdown cells upon e‐MI blockage, inhibition of lysosomal proteases did not increase their intracellular levels, suggesting that lysosomes no longer contributed to the degradation of these mutant proteins once e‐MI is compromised (Fig. 2a,b). It is possible that nonlysosomal proteolysis or extracellular release of tau is activated in response to the e‐MI blockage to prevent its cellular accumulation.

To understand whether failure to undergo e‐MI was due to LE toxicity in these cells or a primary defect in tau targeting and degradation, we presented purified tau proteins (WT, A152T, or P301L) to intact LE previously incubated or not with protease inhibitors. We found that WT tau was taken up and efficiently degraded by LE (Fig. 2c,d). This process was significantly impaired for tau‐P301L and, to higher extent, for tau‐A152T (Fig. 2c,d). Exposure of these purified mutant proteins to disrupted LE shows no difference in their degradation rates compared to WT tau (Fig. S1, Supporting information), suggesting that defective e‐MI occurs at the level of internalization of mutant tau proteins inside LE. These findings suggest that e‐MI can effectively internalize and degrade unmodified forms of tau, but mutations in this protein greatly reduce the ability of LE to contribute to its turnover.

Lastly, to determine the contribution of macroautophagy to the degradation of mutant forms of tau, we silenced Atg7 in the N2a cells expressing the different tau proteins. Macroautophagy blockage resulted in preferential accumulation of A152T, but not WT and P301L tau (Fig. 2e,f). As in the case of cells with compromised e‐MI, although disruption of macroautophagy did not result in accumulation of WT or P301L tau, their rates of lysosomal degradation were markedly reduced. This supports a possible engagement of other clearance mechanism(s) in response to the blockage of macroautophagy.

Overall, these results suggest a switch in the autophagic degradation of tau proteins bearing these pathogenic mutations (Fig. 2g). Thus, the A152T mutation blocks degradation of tau in LE, favors its rerouting toward macroautophagy degradation, and has only a mild effect on its degradation by CMA. In contrast, the P301L mutation is the one more severely affecting tau degradation by lysosomes as, in agreement with its insensitivity to blockage of overall lysosomal proteolysis, we failed to detect significant contribution of CMA, e‐MI, or macroautophagy to its cellular clearance (Fig. 2g).

### Autophagic response to cellular expression of A152T or P301L tau

**Figure 3:**
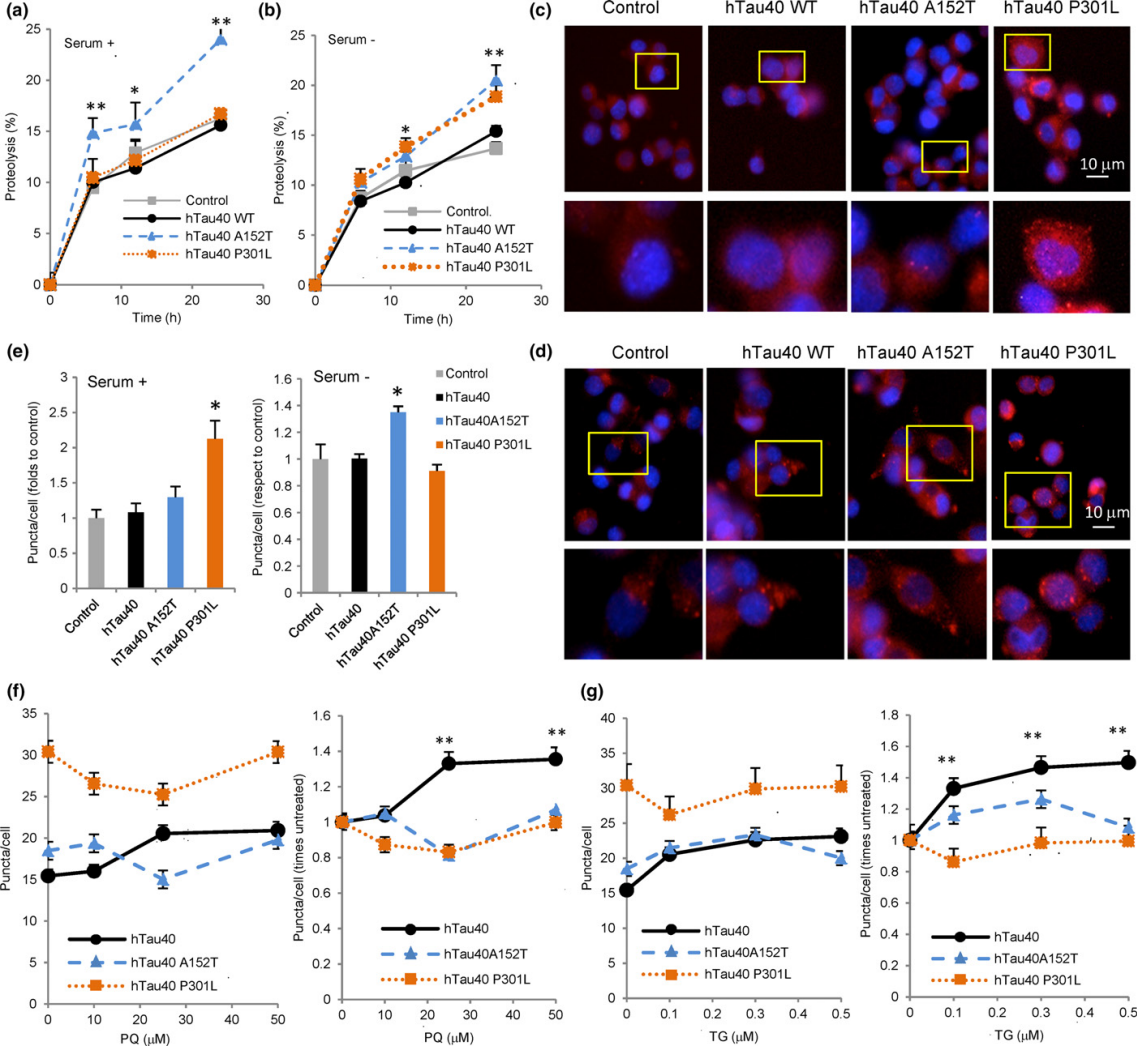
Effect of disease‐related mutant tau proteins on CMA. (a,b) Proteolysis of long‐lived proteins in mouse neuroblastoma cell line Neuro‐2a (N2a) treated with doxycycline to activate expression of the indicated tau proteins. Protein degradation was measured at the indicated times, in cells maintained in the presence (a) or absence (b) of serum, as the amount of acid‐precipitable radioactivity (amino acids and small peptides) released into the media. *n* = 3 different experiments with triplicate wells. (c–g) Same N2a cells as in a, were treated with doxycycline for 72 h and then transduced with lentivirus carrying the KFERQ‐PS‐Dendra2 reporter and maintained in the presence (c) or absence of serum (d) or in the presence of paraquat (PQ) (f) or thapsigargin (TG) (g) at the indicated concentrations. Representative images of cells maintained in the presence (c) or absence (d) of serum 16 h after photoswitching. Insets show higher magnification. Nuclei were stained with DAPI. (e) Quantification of average number of puncta per cell section in experiments as the ones in c and d. (f–g) Quantification of average number of puncta per cell in absolute values (left) or relative to the number in untreated cells (right) in experiments as in c but in the presence of paraquat (f) or thapsigargin (g). *n* > 800 cells/condition in three different experiments with triplicate wells. All values are mean ± SEM. Differences are significant for *P < 0.05 and **P < 0.01.

Pathogenic tau variants can negatively impact the activity of different proteolytic systems. To determine whether the observed changes in autophagic clearance of A152T and P301L tau could be in part a consequence of their toxic effect on these pathways, we analyzed the effect of these mutant proteins on overall protein degradation. We measured proteolysis of long‐lived proteins (preferentially degraded by autophagy) in N2a cells treated with doxycycline to activate expression of the different tau proteins and labeled with [^3^H] leucine for 48 h. Measurements were carried out under basal (Serum +) and inducible conditions (Serum −). Cells expressing tau‐A152T displayed significantly higher rates of intracellular protein degradation than the other cells under basal conditions (Fig. 3a). A similar significant increase in protein degradation was observed in response to serum removal in cells expressing either of the mutants (Fig. 3b). These changes suggest an initial cellular response to the expression of these mutant proteins, whereby cells upregulate intracellular proteolysis likely to sustain proteostasis and facilitate removal of the toxic proteins.

To identify the impact of the tau mutants on each of the autophagic pathways, we first used a photoswitchable reporter for CMA (generated by fusion of the fluorescent protein Dendra with a CMA‐targeting motif (KFERQ‐like), KFERQ‐Dendra (Koga *et al*., 2011)). This fluorescent protein changes its localization from the cytosol into lysosomes when CMA is active, shifting from a diffuse fluorescent cytosolic pattern to a punctate pattern (Fig. S2, Supporting information shows colocalization of single‐color fluorescent CMA reporter (KFERQ‐PA‐mCherry) puncta with a lysosomal marker to confirm proper targeting of the probe to lysosomes in these cells). The number of fluorescent puncta in cells expressing tau‐A152T remained unchanged under basal conditions (Fig. 3c,e), suggesting that CMA was likely not responsible for the protein degradation burst in these cells. We did however find that under serum deprivation conditions, tau‐A152T‐expressing cells displayed significantly higher CMA (Fig. 3d,e; 30% increase) than control cells. Cells expressing tau‐P301L displayed significant upregulation of CMA under basal conditions that was no longer observed upon serum removal (Fig. 3c–e). Overall, these findings suggest that cells react to pathogenic tau by increasing their basal or inducible CMA activity. However, at least in the presence of tau‐P301L, the further increase in CMA normally induced by nutrient deprivation is no longer attainable.

To explore the possibility that pathogenic forms of tau may limit the ability of cells to upregulate CMA in response to stress, we exposed cells expressing the CMA reporter and tau proteins to increasing concentrations of paraquat (to induce oxidative stress) or to thapsigargin (to induce ER stress). Cells expressing WT tau behave as control cells and display a dose‐dependent increase in CMA activity upon exposure to paraquat (Fig. 3f) or thapsigargin (Fig. 3g). However, cells expressing the mutant tau proteins failed to further upregulate CMA and even a decrease was noticeable in cells expressing tau‐P301L (Fig. 3f,g). These results support a general inability of the cells to upregulate their CMA activity to handle additional stress in the presence of A152T or P301L tau. This inability to accommodate the autophagic response to the stressor may contribute to the higher sensitivity to both paraquat and thapsigargin that we observed in cells expressing either of the two mutant proteins (Fig. S3, Supporting information).

**Figure 4:**
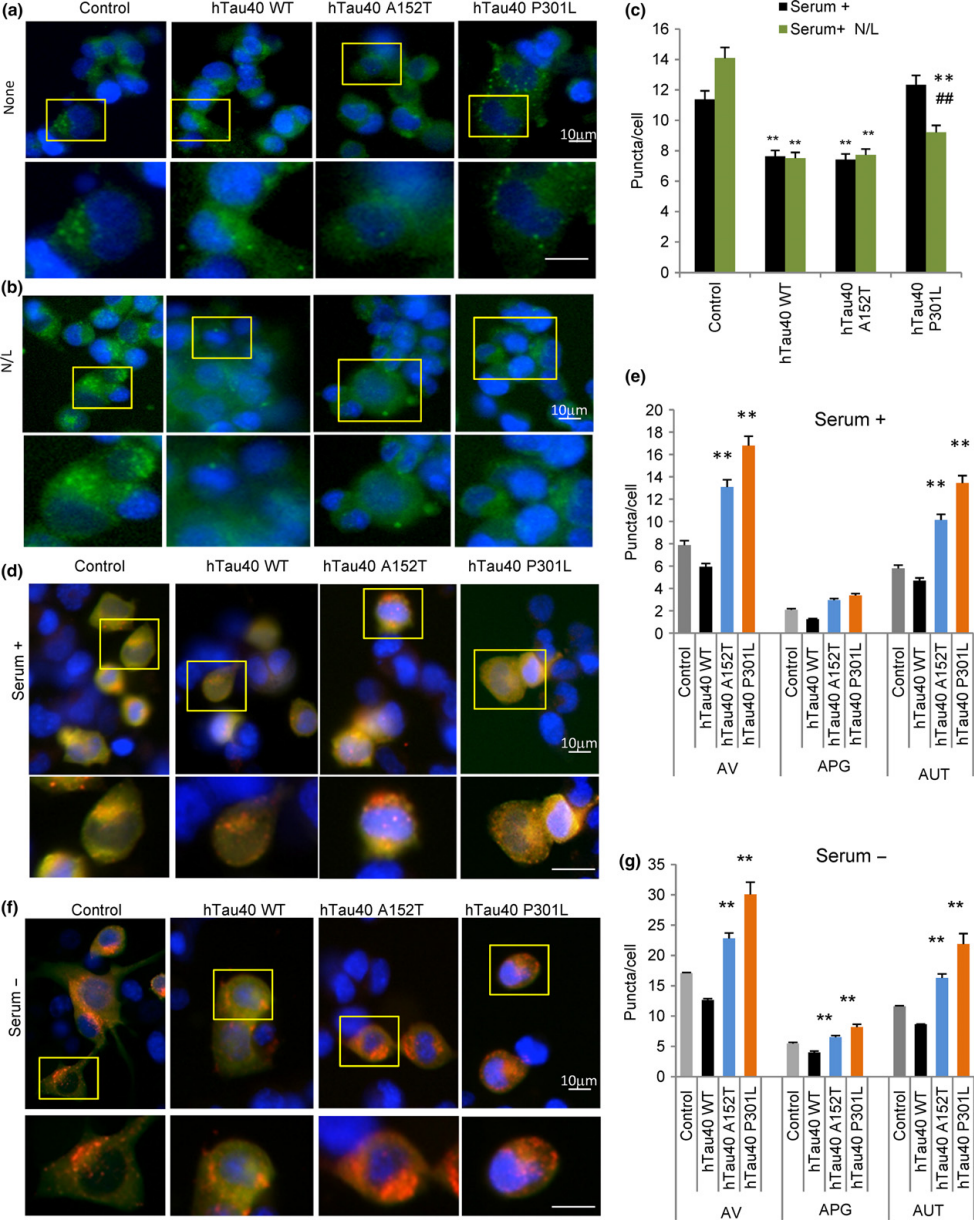
Effect of disease‐related mutant tau proteins on different autophagic pathways. (a,b) Representative images of mouse neuroblastoma cell lines Neuro‐2a (N2a) treated with doxycycline to activate expression of the indicated tau proteins and transduced with lentivirus carrying the N‐KFERQ‐mVenus and C‐KFERQ‐mVenus. Cells were incubated without additions (a) or in the presence of NH_4_Cl 20 mm and leupeptin 100 μm (N/L) for 4 h (b). Insets show higher magnification. Nuclei were stained with DAPI. (c) Quantification of average number of puncta per cell section in experiments as the ones in a and b. *n* > 800 cells/condition in three different wells. (d, f) Representative images of N2a treated as in a, but transduced with lentivirus carrying the mCherry‐GFP‐LC3 reporter and maintained in the presence (d) or absence (f) of serum. Nuclei are highlighted with DAPI in blue. Insets show higher magnification images. (e,g) Quantification of total number of autophagic vacuoles (AV), autophagosomes (APG, yellow puncta) and autolysosomes (AUTL, only red puncta) in cells maintained in the presence (e) or absence (g) of serum. *n* > 800 cells/condition in three experiments with triplicate wells. All values are mean ± SEM. Differences with untreated (#) or with control (*) were significant for *,#*P* < 0.05 and **,##*P* < 0.01.

Our *in vitro* study revealed that despite lack of internalization/degradation, a fraction of the mutant tau proteins still associated with the membrane of LE (Fig. 2c). Therefore, we next analyzed possible changes in e‐MI activity in these cells using a reporter developed to study this pathway in flies and modified by our laboratory for use in mammalian cells (Uytterhoeven *et al*., 2015). We transduced cells with the split mVenus plasmid containing the N‐terminal and C‐terminal of this protein tagged to the KFERQ motif (N‐KFERQ‐mVenus and C‐KFERQ‐mVenus). Fluorescence is only observed when these two constructs are in very close proximity within the multivesicular bodies that form during e‐MI. Any hemi‐KFERQ‐mVenus targeted to lysosomes would not fluoresce as it will be unfolded and rapidly degraded. As expected, fluorescence puncta were visible in transduced control cells (Fig. 4a) and blockage of endo/lysosomal degradation with ammonium chloride and leupeptin significantly increased the number of fluorescent puncta by preventing their degradation (Fig. 4b). In the presence of any of the tau proteins, we found some sequestration of the probe in the multivesicular bodies, albeit significantly less in cells expressing the WT and A152T protein. However, the probe content did not increase upon treatment with protease inhibitors, suggesting that its internalization/degradation through this pathway was halted (Fig. 4b,c). It is possible that inhibition of e‐MI by WT tau is due to competition of tau with other substrates for this pathway, including the probe. However, a direct inhibitory effect of tau bound to the LE membrane is a more likely scenario in the case of the mutant proteins, as none of them undergo detectable degradation through e‐MI but still associate with LE membranes (Fig. 2c).

Lastly, to understand whether either of these point mutations of tau modifies MA activity, we monitored MA by transducing cells with a well‐characterized component of autophagosomes (Kabeya *et al*., 2000), the light chain 3 protein (LC3) fused to mCherry and GFP. The tandem mCherry‐GFP‐LC3 reporter (Kimura *et al*., 2007) allows to measure autophagosomes and autolysosomes (as yellow and red puncta, respectively). This is because the fluorescence of GFP is quenched once it reaches the acidic environment of the lysosomal lumen and, thereby, only red fluorescence is observed. We found that abundance of autophagic vacuoles (autophagosomes + autolysosomes) significantly increased in cells expressing either of the two tau mutants (Fig. 4d,e). This increase was mainly due to higher content of autolysosomes (red puncta) (Fig. 4d,e), in support of increased macroautophagic flux. To discard possible cell‐type‐dependent effects, we transiently transfected a second line of neuronal‐like cells (SH‐SY5Y) with the different tau mutants and confirmed a similar increase in macroautophagy flux using immunoblot for LC3‐II in cells treated or not with lysosomal protease inhibitors (Fig. S4, Supporting information). The significantly higher abundance of autophagic vacuoles was still evident in cells expressing the two tau mutants when serum was removed from the culture media, suggesting that macroautophagy is still upregulated under these conditions (Fig. 4f,g). However, in this case, a significant increase in autophagosomes and a reduction in autolysosomes become evident when compared to basal conditions. This could be indicative of some incipient saturation of the lysosomal system for autophagosome clearance. Although tau‐P301L is not primarily degraded by macroautophagy, the observed increase in the macroautophagy flux may be a cellular response to this pathogenic form of tau. In fact, increased macroautophagy may be responsible for the increase in the degradation of long‐lived proteins that we observed for both mutant forms of tau under these conditions (Fig. 3b). As both CMA and macroautophagy are stress‐induced forms of autophagy, we also analyzed changes in macroautophagy in cells expressing WT, A152T, and P301L subjected to additional stressors. As in the case of CMA, the presence of the pathogenic proteins limited the ability of macroautophagy to become upregulated upon oxidative or ER stress (Fig. S5, Supporting information).

In summary, when comparing the pathogenic tau mutation P301L with the risk‐associated mutation A152T, we found that both reduced normal turnover of tau by autophagy, but that the effect of the P301L mutation was more pronounced (summarized in Fig. 2g and Fig. S6, Supporting information). The most notable difference between the two tau mutants was the inability of P301L to undergo degradation by CMA or by macroautophagy. Conversely, cells reacted to the presence of both mutant proteins by upregulating basal protein degradation, mainly through macroautophagy, but this constant activation seems to limit any further upregulation to accommodate to additional stress thus increasing their vulnerability to stressors.

### Impact of tau modifications on its degradation by selective autophagy

Our previous studies and data presented in this work support substantial contribution of CMA to the degradation of wild‐type unmodified tau (Wang *et al*., 2009). Interestingly, although judging by the studies in intact cells the contribution of e‐MI to tau degradation is small (Fig. 2a), our *in vitro* studies with isolated LE revealed a high efficiency for e‐MI of tau (Fig. 2c). This suggests that in some cellular conditions or upon specific tau modifications, this could become an effective way for tau degradation. To further explore modifications in tau that may impact its degradation by CMA and e‐MI, we next analyzed the degradation of different tau isoforms and tau mutations that change its biochemical properties (e.g., aggregation, oxidation, or pseudophosphorylation).

**Figure 5:**
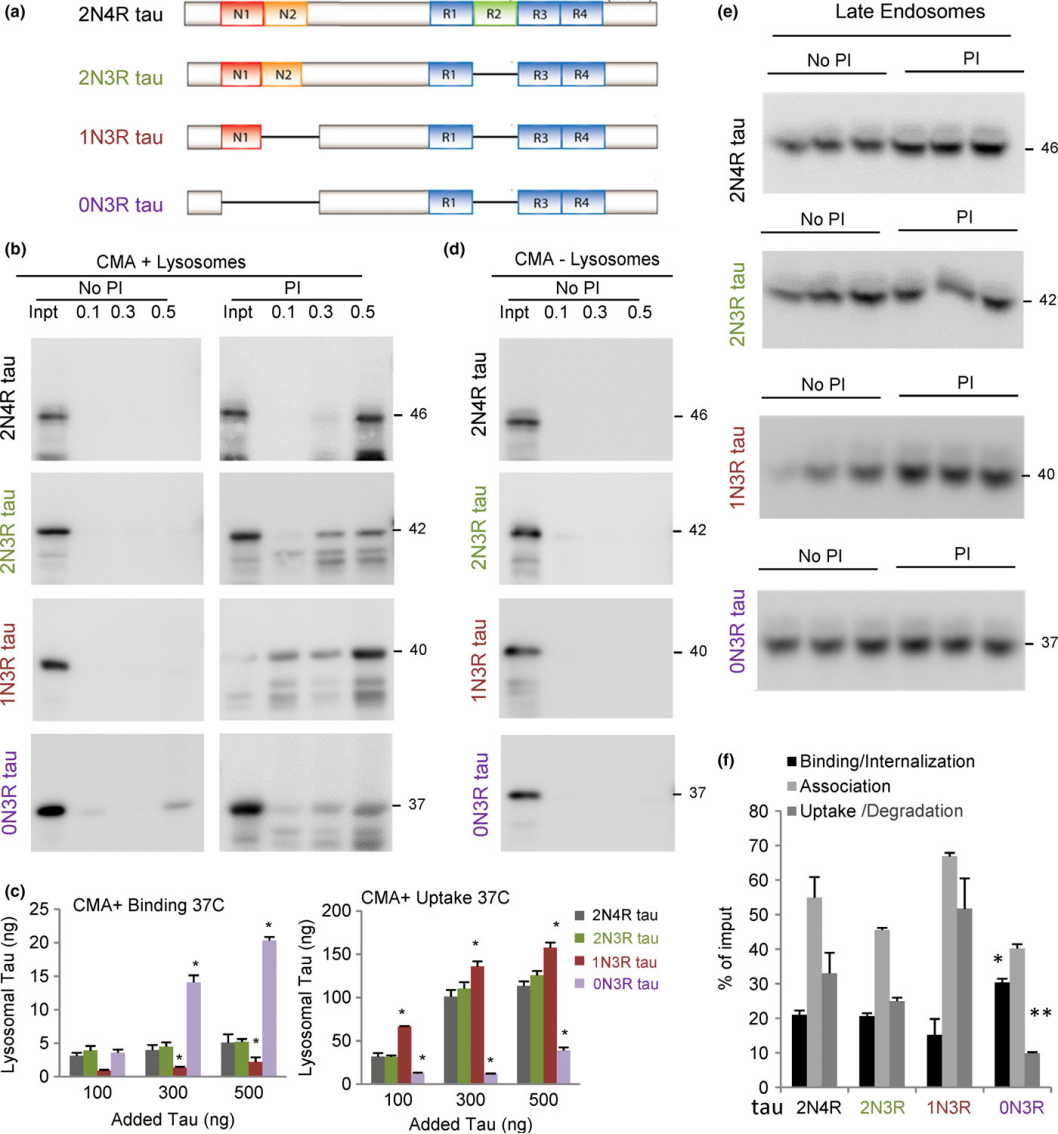
Degradation of different tau isoforms by selective autophagic pathways. (a) Scheme of the domain composition of the different tau isoforms analyzed in this study. (b) Immunoblots for tau of isolated CMA‐active lysosomes pretreated or not with protease inhibitors (PI) for 10 min at 4 °C and then incubated with the indicated concentrations (μg) of tau proteins at 37 °C for 20 min. Inpt: input. (c) Quantification of binding (left) and uptake (right) of tau proteins by the CMA‐active lysosomes. Values are indicated in ng, calculated from the densitometric quantification of a known amount of purified protein. *n* = 5. (d) Immunoblot of tau proteins incubated under the same condition as in b but with CMA‐inactive (−) lysosomes. (e) Immunoblots for tau in isolated late endosomes pretreated or not with protease inhibitors (PI) for 10 min at 4 °C and then incubated with the indicated tau proteins (0.5 μg) at 37 °C for 30 min. (f) Quantification of binding, association, and uptake/degradation of tau proteins by the late endosomes. Values are indicated as percentage of the input, calculated from the densitometric quantification of a known amount of purified protein. *n* = 4. All values are mean ± SEM. Differences with hTau40 WT were significant for *P < 0.05 and **P < 0.001.

We used four tau isoforms with different number of N and R domain: 2N4R tau (referred to as hTau40 in the rest of the study), 2N3R tau, 1N3R tau, and 0N3R tau (Fig. 5a). Analysis of their uptake by isolated CMA‐active lysosomes revealed that 2N3R tau behaved similarly to 2N4R tau (which we have used in the rest of the study as control). This supports that the second R domain has little impact on CMA of tau (Fig. 5b,c). Absence of the second N‐terminal insert (in 1N3R tau) did not reduce CMA of tau, but instead this isoform displayed faster internalization (lower binding because of more efficient uptake) (Fig. 5b,c). In contrast, once the first N‐terminal insert is lost (in 0N3R tau), we observed a very pronounced decrease in tau uptake (Fig. 5b,c). As in the case of A152T, the enhanced binding of 0N3R tau to lysosomes did not result from nonselective interaction with membranes, because we did not observe binding of 0N3R tau to CMA‐inactive lysosomes (Fig. 5d). These results suggest that the second N‐terminal insert plays a crucial role in the uptake of tau into the lysosomal lumen, but it is not required for hsc70 binding and lysosomal targeting of tau. These findings are consistent with the fact that the KFERQ‐like motifs in tau are in the C‐terminal domain. Absence of the second N‐terminal insert also significantly reduced e‐MI of tau (Fig. 5e,f).

**Figure 6:**
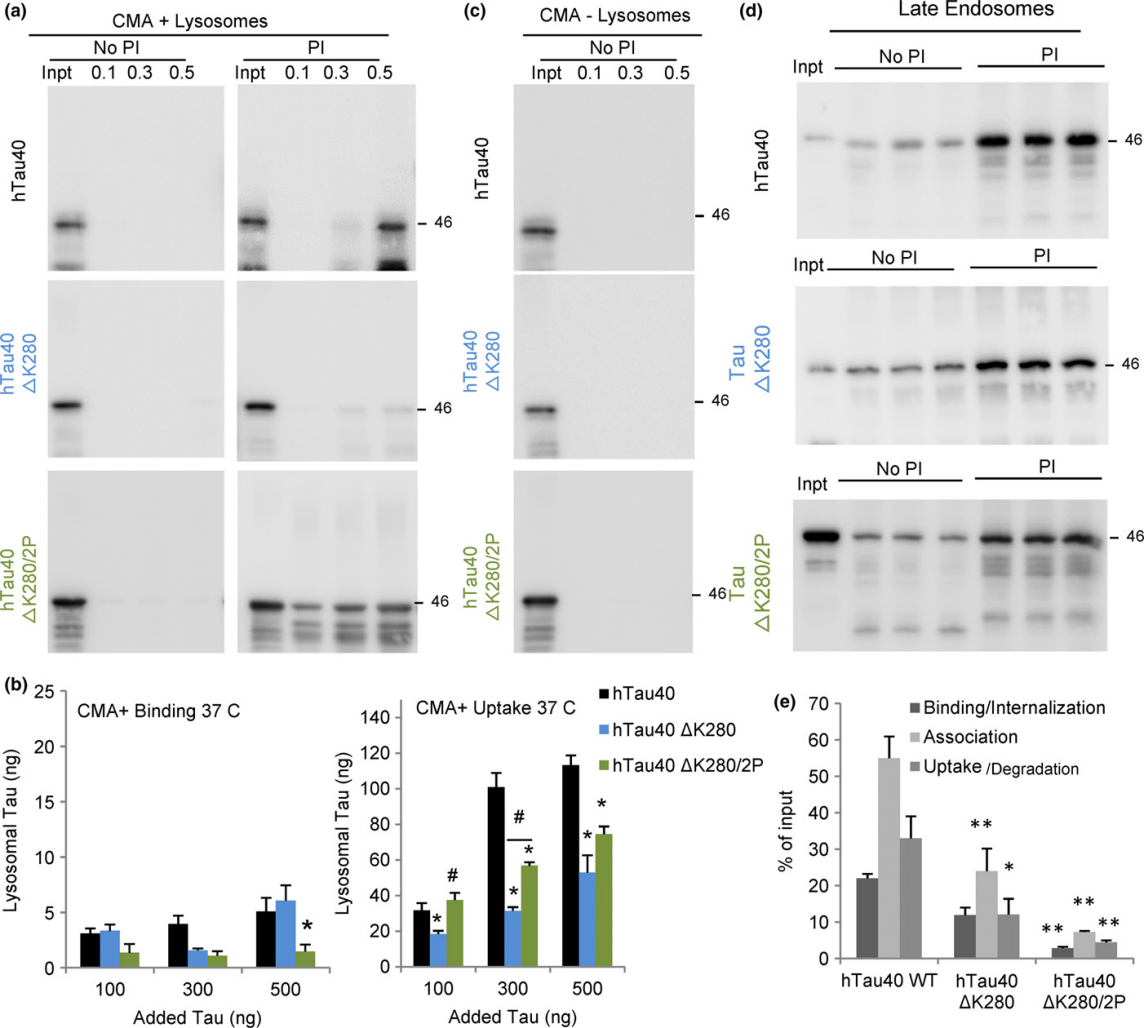
Degradation of aggregate‐prone mutant tau (hTau40 ΔK280) by selective autophagic pathways. (a) Immunoblots for tau of isolated CMA‐active lysosomes, pretreated or not with protease inhibitors (PI) for 10 min at 4 °C and then incubated with the indicated concentrations of wild‐type tau (hTau40), tau with lysine 280 deleted (hTau40 ΔK280) or with lysine 280 replaced with two proline residues to disrupt β propensity (hTau40 ΔK280/2P) at 37 °C for 30 min. Inpt: input. (b) Quantification of binding (left) and uptake (right) of the tau proteins by the CMA‐active lysosomes. Values are indicated in ng, calculated from the densitometric quantification of a known amount of purified protein. *n* = 4. (c) Immunoblot of tau proteins incubated under the same condition as in b but with CMA‐inactive (‐) lysosomes. (d) Immunoblots for tau in isolated late endosomes pretreated or not with protease inhibitors (PI) for 10 min at 4 °C and then incubated with the indicated tau proteins (0.5 μg) at 37 °C for 30 min. (e) Quantification of binding, association, and uptake/degradation of tau proteins by the late endosomes. Values are indicated as percentage of the input, calculated from the densitometric quantification of a known amount of purified protein. *n* = 3. All values are mean ± SEM. Differences with hTau40 WT (*) or between the mutants (#) were significant for *,#*P* < 0.05 and **,*P* < 0.01.

Interestingly, a deletion of lysine 280 (hTau40 ΔK280), known to lead to tau aggregation (Khlistunova *et al*., 2006), turned this protein into a very poor CMA substrate (Fig. 6a,b). We did not detect enhanced binding of this tau mutant to CMA‐active or CMA‐inactive lysosomes (Fig. 6a–c). This finding rules out that diminished translocation could be due to oligomerization or aggregation of this mutant at the surface of lysosomes, which was previously described to be the case for pathogenic proteins such as α‐synuclein or LRRK2 (Cuervo *et al*., 2004; Orenstein *et al*., 2013). Although the tendency of hTau40 ΔK280 to aggregate could be the determinant of its poor clearance by CMA, we propose a direct effect of this mutation on tau's ability to undergo degradation through CMA, as inefficient CMA of hTau40 ΔK280 seems, in part, independent of aggregation. Thus, insertion of two prolines in the ΔK280 background (hTau40 ΔK280/2P), which prevents tau aggregation (Barghorn & Mandelkow, 2002), only partially rescued CMA uptake of the ΔK280 mutant (Fig. 6a,b). This suggests that in addition to the second N‐terminal insert of tau, its tertiary structure is also important for tau's lysosomal internalization, because the two prolines in hTau40 ΔK280/2P act as beta‐structure breakers. This conformational change, but not the aggregation itself, interferes also with tau degradation by e‐MI, as we found a significant decrease in the uptake of both mutants by LE (Fig. 6d,e). Because reduced CMA and e‐MI of these mutants did not associate with higher amount of protein bound to the lysosomal or LE membrane, we propose that the defect in this case is not at the level of internalization but possibly at its selective targeting to these compartments.

**Figure 7:**
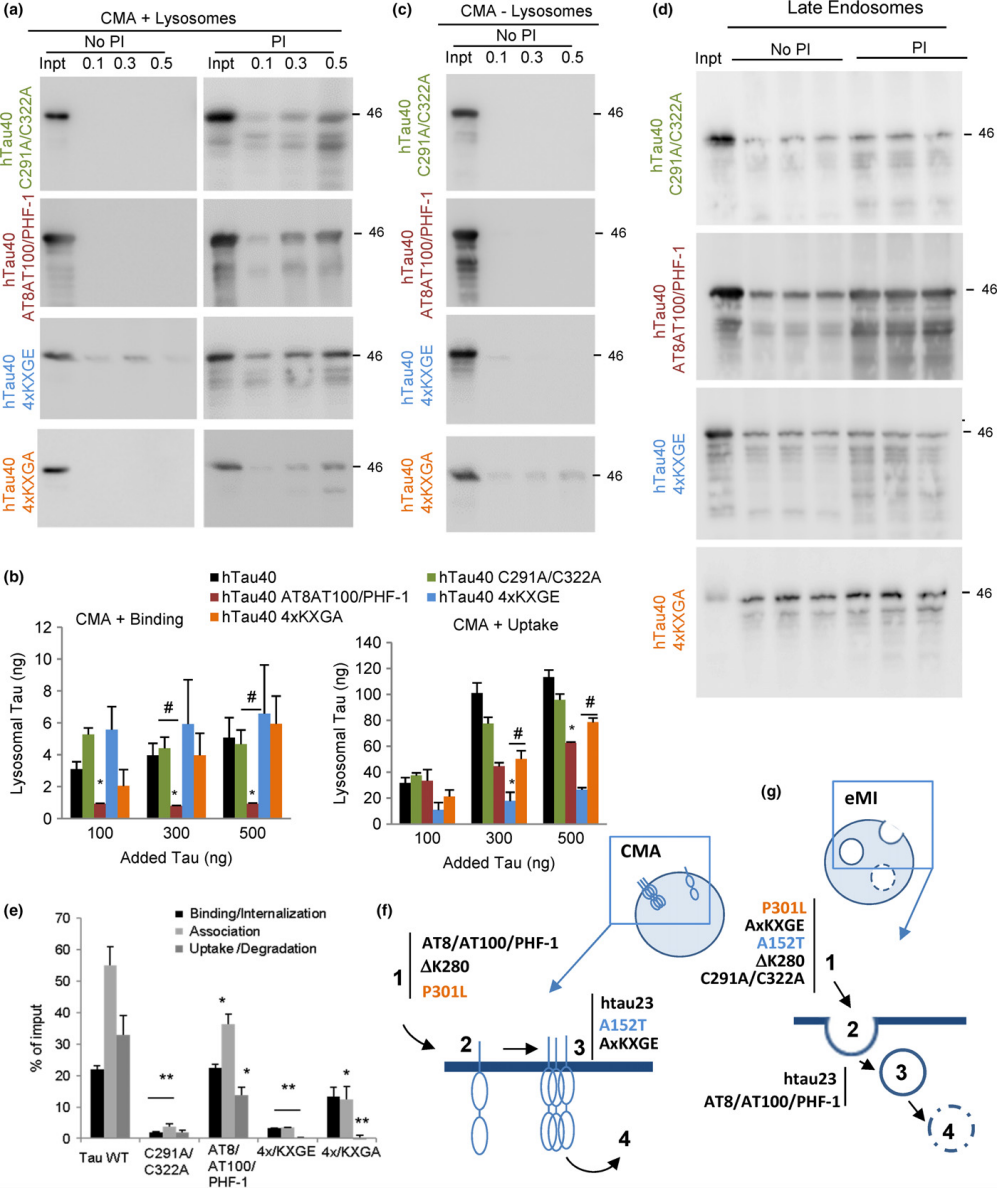
Effect of oxidation and pseudophosphorylation on the degradation of tau by selective autophagic pathways. (a) Immunoblots for tau of isolated CMA‐active lysosomes, pretreated or not with protease inhibitors (PI) for 10 min at 4 °C and then incubated with tau with cysteine 291 and 322 replaced by alanine (hTau40 C291A/C322A), or with mutations S199E+S292E+T214E (AT8 site), plus T212E+S214E (AT100) plus S396E+S404E (PHF‐1) (to yield htau40 AT8/AT100/PHF‐1), with serine 262, 293, 324, and 356 replaced by glutamic acid (4xKXGE) (to mimic hyperphosphorylation) or with alanine (4xKXGA) (to disrupt phosphorylation). Proteins were added at the indicated concentrations and incubations were performed at 37 °C for 20 min. Inpt: input. (b) Quantification of binding (left) and uptake (right) of the tau proteins by the CMA‐active lysosomes. Values are indicated in ng, calculated from the densitometric quantification of a known amount of purified protein. *n* = 3. (c) Immunoblot of tau proteins incubated under the same condition as in b but with rat CMA‐inactive (−) lysosomes. (d) Immunoblots for tau in isolated rat late endosomes pretreated or not with protease inhibitors (PI) for 10 min at 4 °C and then incubated with the indicated tau proteins (0.5 μg) at 37 °C for 30 min. (e) Quantification of binding, association, and uptake/degradation of tau proteins by the late endosomes. Values are indicated as percentage of the input, calculated from the densitometric quantification of a known amount of purified protein. *n* = 3. All values are mean ± SEM. Differences with hTau40 WT (*) or between the mutants (#) were significant for *P < 0.05 and **P < 0.001. (f) Scheme of the steps of CMA disrupted for each of the indicated tau variants. 1. Targeting; 2. binding; 3. internalization; and 4. degradation. (g) Scheme of the steps of e‐MI disrupted for each of the indicated tau variants. 1. Targeting; 2. binding; 3. internalization; and 4. degradation.

Lastly, we analyzed the impact of two different posttranslational modifications, oxidation and phosphorylation, on CMA and e‐MI of tau. We did not detect differences with WT tau on binding or uptake by lysosomes of the hTau40 C291A/C322A mutant that renders the protein unable of becoming oxidized thus reducing its aggregation propensity (Mukrasch *et al*., 2009) (Fig. 7a,b). This lack of effect could be due to the fact that incubation was not performed under pro‐oxidizing conditions, when disulfide bonds could form between cysteines. However, in these same conditions, we found a significant decrease in the association and internalization of the hTau40 C291A/C322A mutant with LE by e‐MI (Fig. 7d,e). Previous studies have demonstrated that oxidized proteins accumulate inside multivesicular bodies (Cannizzo *et al*., 2012), suggesting that oxidation may be a prerequisite to complete internalization of tau by e‐MI, and that the LE environment may contribute to that modification.

Phosphorylation has been extensively studied in the context of tau pathology (Mandelkow *et al*., 1995; Wang & Mandelkow, 2016), but due to the multiplicity of phosphorylation sites in the protein, the site‐specific impact of phosphorylation on tau biology is not well understood. We chose to analyze the effect of mutations that mimic phosphorylation at specific residues that form part of the epitopes of antibodies elevated in AD or for which an aggregation propensity has been described (Biernat & Mandelkow, 1999; Chang *et al*., 2011; Combs *et al*., 2011; Kiris *et al*., 2011; Prokopovich *et al*., 2017, 50). We used S to E mutations that mimic phosphorylation in the KXGS motifs of the repeat domain that controls microtubule binding and tau aggregation rate (Fischer *et al*., 2009). Exchanging S^262^, S^293^, S^324^, and S^356^ for E (hTau40 4xKXGE) was used to generate the pseudophosphorylated tau, while the 4xKXGA mutant was used as a nonphosphorylatable control. The second pseudophosphorylation form of tau selected for this study was htau40 AT8/AT100/PHF‐1. The nomenclature is based on the phospho‐epitopes recognized by antibodies raised against tau from AD brains (AT8, AT100, or PHF‐1) when tau is phosphorylated at specific sites at either flanking domain of the repeats (Bibow *et al*., 2011). The two pseudophosphorylated tau proteins (hTau40 AT8/AT100/PHF‐1 and hTau40 4xKXGE) displayed significantly decreased lysosomal uptake when compared to hTau40 WT Tau (Fig. 7a,b). Interestingly, the step of CMA affected in each pseudophosphorylation mutant seemed different. In the case of hTau40 AT8/AT100/PHF‐1, defective lysosomal uptake originates mostly from inefficient binding of this protein to lysosomes. However, in the case of hTau40 4xKXGE, which leads to a more severe CMA defect, binding to the lysosomal membrane still occurred, but translocation was severely impaired (Fig. 7a,b). Nonselective binding to membranes of CMA‐incompetent lysosomes was not detected for any of these proteins (Fig. 7c). Changing glutamic acid for alanine (hTau40 4xKXGA), which serves as a control for the pseudophosphorylation, partially rescued the uptake of this form of tau (Fig. 7a,b). Analysis of e‐MI of the pseudophosphorylated forms of tau using LE showed reduced uptake/degradation of hTau40 AT8/AT100/PHF‐1 when compared to WT tau (Fig. 7d,e). This effect was more pronounced in the case of hTau40 4xKXGE, whose uptake and degradation by LE was completely abolished (Fig. 7d,e), suggesting that phosphorylation in the microtubule‐binding domain diminishes its degradation by e‐MI. As in the case of CMA, only partial rescue was observed in the hTau40 4xKXGA mutant (Fig. 7d,e). This argues that changes, other than phosphorylation, in that region may have a disruptive effect on the selective sequestration of tau by LE.

Overall, these studies have identified modifications in tau with a negative impact on its degradation by different autophagic pathways and have discriminated which forms of tau exert a toxic effect on its own degradation systems. A summary of the changes in CMA and e‐MI of the different tau variants is provided in Fig. 7 (Supporting information) and a schematic of the steps affected in these pathways in Fig. 7f,g.

## Discussion

In this work, we found that modifications in the amino acid sequence of tau affect its normal degradation through CMA and e‐MI and that in turn, some of these modifications can have an impact on the activity of these degradation pathways (summarized in Figs 2g and 7f,g and Figs S6 and S7, Supporting information). Understanding differences in this dual interplay between different forms of tau and the autophagic pathways may help to discover why some tau modifications contribute to pathogenicity while others only increase disease risk (Wang & Mandelkow, 2016). Similarly, in light of the extensive repertoire of posttranslational modifications already described for this protein (Prokopovich *et al*., 2017), a systematic analysis of the impact of these modifications on different aspects of tau biology, including its turnover by selective forms of autophagy as investigated in this study, should help to clarify the physiological regulation of tau and the basis of its toxicity in the aging brain.

The P301L tau mutation lies within the repeated domain and enhances tau aggregation, and its role in FTD pathogenesis has been well characterized. By contrast, the A152T mutation lies far outside the repeat domain and has only a weak effect on tau aggregation, and its role as a risk factor in FTD spectrum disorders or AD is still poorly understood. The different effects of both mutations have been further demonstrated by comparing the phenotype of P301L (Lin *et al*., 2003) and the novel tau‐A152T transgenic mouse models (Decker *et al*., 2016; Maeda *et al*., 2016; Sydow *et al*., 2016). However, the basis for these phenotypic differences is not fully understood. In this work, we have found that the P301L mutation interferes with the degradation of tau through all the autophagic pathways. In contrast, the A152T mutation on tau disrupts its degradation by e‐MI but has a considerably less pronounced impact on the degradation of this protein by CMA, which is one of the preferred pathways for tau degradation under physiological conditions (summarized in Fig. S6, Supporting information). The different impact of both mutations in the autophagic degradation of tau goes in line with the severity of tau toxicity in the mouse models expressing copies of these proteins (Lin *et al*., 2003; Decker *et al*., 2016; Maeda *et al*., 2016; Sydow *et al*., 2016). Pathology and tau aggregation in mouse expressing the Tau‐A152T mutation occur at a slower pace than in the tau‐P301L overexpressor, where tangles appear as early as 4.5 month (Lin *et al*., 2003) and where we found a more severe impairment of its autophagic degradation.

Interestingly, the marked increase in degradation of Tau‐A152T by macroautophagy (which we propose to be compensatory to its reduced degradation by CMA) was not observed for the P301L‐tau mutant. This failure to reroute P301L‐tau to macroautophagy was not due to a toxic effect of the pathogenic protein on macroautophagy, as we found that expression of this mutation induces an increase in macroautophagy flux. This suggests that cells in the presence of this toxic protein try to compensate and increase their degradation capacity. Whether the failure to degrade P301L through macroautophagy, despite the upregulation of this system, is the result of problems in the recognition/targeting of this mutant protein toward this pathway requires future investigation. However, independently of the mechanism, the upregulation of macroautophagy did not increase P301L‐tau degradation by this pathway nor did it reduce the cytotoxicity of this protein. This poses possible limitations to the therapeutic value of mere upregulation of macroautophagy in this context. In contrast, the effective degradation of the A152T mutant by macroautophagy (which we attribute to its reduced degradation via CMA) suggests that modulation of macroautophagy may be a suitable alternative to reduce intracellular abundance of Tau‐A152T protein.

We found that e‐MI did not contribute to degradation of either tau‐A152T or tau‐P301L, and that neither protein could be targeted to LE for degradation, not even *in vitro*. This is interesting because we initially attributed the lack of intracellular accumulation of tau‐P301L, despite its inability to undergo degradation by any of the autophagic pathways, to possible release of tau‐P301L from the cell. In this respect, we considered that tau‐P301L could be released upon delivery to LE, from where it could be secreted into exosomes. However, intracellular levels of tau‐P301L are not affected by disruption of multivesicular body/exosome formation. It is possible that instead of our proposed release of tau‐P301L via LE and exosomes, other systems contribute to tau‐P301L secretion or that other proteolytic systems (i.e., the ubiquitin/proteasome system) contribute to its breakdown.

In our experimental setting, the observed reduction in CMA activity and basal upregulation of macroautophagy in cells expressing the mutant proteins does not seem due to changes in lysosomal biogenesis because the number of endo/lysosomal compartments (positive for LAMP1) remained unchanged. However, we cannot discard that once in a more chronic setting, indirect effect of mutant tau proteins on lysosomal biogenesis may also occur. Despite this basal compensation between CMA and macroautophagy, the fact that cells expressing either of the tau mutant proteins fail to further upregulate these autophagic pathways in response to additional stressors suggests that they might be functioning at the limit and could be close to exhaustion. This effect may be more pronounced in cells expressing P301L‐tau which had consistently higher basal upregulation of macroautophagy. This inability to adapt the autophagic response to stressors would be particularly worrisome in the context of a chronic disease in the aging brain where a failure of autophagy might trigger faster disease progression. In fact, defective macroautophagy has been extensively reported in FTD and other tauopathies (Nixon, 2013).

Targeting of proteins for degradation by CMA and e‐MI requires a pentapeptide motif recognized by the hsc70 chaperone (Sahu *et al*., 2011; Kaushik & Cuervo, 2012). Tau bears two of such motifs in its C‐terminal region which are required for its CMA targeting (Wang *et al*., 2009). The comparative analysis presented here with four different tau isoforms supports that in addition to this targeting motif, the N‐terminal inserts are crucial for the degradation of tau through CMA, and to a lesser extent through e‐MI. While the KFERQ‐like motif in the C‐terminal domain is required for targeting, the internalization step of both CMA and e‐MI step is disrupted when the N‐terminal insert is missing. It is still unclear how this domain facilitates internalization of tau, as our previous data suggested a ‘C‐terminus first’ entry through CMA (Wang *et al*., 2009). It is possible that interaction of the N‐terminal insert with components of the lysosomal membrane contributes to stabilize tau during the internalization process. In fact, according to the ‘paperclip model’ (based on FRET pairs) (Jeganathan *et al*., 2006), the N‐ and C‐termini come into close vicinity.

The two phosphomimetic forms of tau tested in this study (mimicking phosphorylation sites within the repeats or in the flanking regions) display defects in their CMA and e‐MI degradation. This suggests that, despite the known limitations of the use of amino acids as phosphorylation mimetics, posttranslational modifications in these tau residues could have a similar negative impact on tau degradation through these two selective forms of autophagy. Notably, the steps of CMA and e‐MI affected by tau phosphorylation are different depending on the phosphorylated regions. When tau is phosphorylated within the microtubule‐binding domain (KXGS motifs), binding to lysosomes occurs, but translocation is disrupted. By contrast, hyperphosphorylation in the flanking domains (AT8 or PHF‐1 epitopes) severely reduces the binding of tau to lysosomes (summarized in Fig. S7, Supporting information). Intriguingly, the effects on e‐MI of both types of phosphorylated tau were opposite: phosphorylation in the microtubule‐binding site reduced LE binding whereas phosphorylation outside this region did not interfere with binding but disrupted internalization. Furthermore, reverting the pseudophosphorylation within the microtubule‐binding domain partially rescued lysosomal uptake of tau, but had a negligible effect on its impaired e‐MI degradation. These findings make appealing the idea that under physiological conditions phosphorylation could be a signal for the triage of tau between CMA and e‐MI. It is plausible that phosphorylation at certain sites can reduce the ability of hsc70 to bind to tau and deliver it to these autophagic compartments. Further analysis of the interaction of hsc70 and its different co‐chaperones with phosphorylated tau should help to elucidate how targeting occurs in those cases in which only internalization or translocation of the phosphorylated proteins is blocked.

The *in vitro* systems with isolated lysosomes and late endosomes have allowed us to directly analyze the impact of the mutations and modifications on the degradation through those compartments, without worrying about additional modifications that could occur when tau is in the cellular context or about the indirect contribution of other proteolytic systems. Combination of *in vitro* and cultured cells studies, as we did for the two FTD‐related mutants, will be required in the future for the other modifications to better understand the consequences of their failure to undergo degradation by these selective pathways. We have selected N2a cells as experimental model to start understanding the complex interplay of tau proteins with autophagic pathways. Studies from our laboratories and others, with pathogenic proteins such as alpha‐synuclein, support that the mechanism of their toxic effect on autophagy is the same independent of the cell type (Cuervo *et al*., 2004). However, future studies in transgenic mouse models expressing these tau variants are needed to confirm whether the toxic effect of P301L‐tau and A152T‐tau on autophagy that we describe here in N2a cells also occurs in these mouse neurons.

Overall, our studies unveil a complex interplay between the multiple modifications of tau and selective forms of autophagy, suggesting that some of these modifications may play regulatory roles in the physiological clearance of tau through these pathways whereas others interfere with normal mechanisms for tau clearance. Understanding these differences between physiological and toxic modifications of tau and the effect of these modified forms of tau in the normal functioning of different forms of autophagy will be key for future development of therapeutic strategies aiming at reducing tau toxicity by modulating its autophagic clearance.

## Experimental procedures

### Animals and cells

Adult male Wistar rats (Charles River Laboratories, Wilmington, MA, USA) were used for subcellular fractionation under institutional approved animal protocols. Where indicated, rats were starved for 48 h to activate CMA. The mouse neuroblastoma cell line Neuro‐2a (N2a) was generated as described before (Khlistunova *et al*., 2006). Cells stably knocked down for LAMP‐2A, Atg7 were generated as described previously (Massey *et al*., 2006) using lentiviral‐delivered small hairpin RNA (shRNA). Cells stably knocked down for Vps4A/B were generated by similar procedures but using the following shRNA from the Mission‐Sigma library (Sigma‐Aldrich, San Luis, MO, USA) VPS4A (TRCN0000101417) and VPS4B (TRCN0000101821). Transient knockdown was performed with RNA interference (RNAi) by transfecting cells with 10 nm of the RNAi duplexes using Lipofectamine RNAiMAX Transfection Reagent (Invitrogen, Carlsbad, CA, USA) in Opti‐MEM media without antibiotics. Efficiency of protein knockdown was tested at 24‐ and 48‐h time intervals post‐transfection by immunoblot analysis. Cells were maintained in Dulbecco's modified Eagle's medium (DMEM) (Sigma), in the presence of 10% fetal bovine serum (FBS), 50 μg mL^−1^ penicillin, and 50 μg mL^−1^ streptomycin at 37 °C with 5% CO_2_.

### Chemicals

Sources of reagents and chemicals were as described before (Kiffin *et al*., 2004; Massey *et al*., 2006; Bandyopadhyay *et al*., 2010). The antibody against mouse LAMP‐2A was developed in our laboratory (Cuervo & Dice, 1996). The antibody against total tau (DA9) was a gift from Dr. P. Davies (The Feinstein Institute, Manhasset, NY, USA). The antibody against Vps4 was from Sigma, against GAPDH from Abcam, against light chain 3 protein (LC3) and Atg7 from Cell Signaling (Danvers, MA, USA), against actin from Stressgen, and against hsc70 from Novus.

### Isolation of subcellular fractions

Lysosomes with high activity for CMA were isolated from rat liver by centrifugation of a light mitochondrial–lysosomal fraction in a discontinuous metrizamide density gradient by the modified method described previously (Cuervo *et al*., 1997). Isolation of late endosomes was performed by centrifugation of a mitochondrial–lysosomal–endosomal fraction in two consecutive continuous Percoll gradients laid over a 2.5 m sucrose cushion using a modified method described before (Castellino & Germain, 1995).

### Intracellular protein turnover

To measure degradation of long‐lived proteins, confluent cells were labeled with ^3^H‐leucine (2 μCi mL^−1^) for 48 h at 37 °C, transfected with the indicated plasmids, and then extensively washed and afterward maintained in complete (10% FBS) or serum‐deprived media containing an excess of unlabeled leucine (2.8 mm) to prevent reutilization of radiolabeled leucine (Kaushik & Cuervo, 2009). Aliquots of the media taken at different times were precipitated with TCA and proteolysis was measured as the percentage of the initial acid‐insoluble radioactivity (protein) transformed into acid‐soluble radioactivity (amino acids and small peptides) at the end of the incubation. Total radioactivity incorporated into cellular proteins was determined as the amount of acid‐precipitable radioactivity in labeled cells immediately after washing.

### Purification of recombinant tau proteins

Recombinant Tau proteins were prepared as described previously (Barghorn *et al*., 2005). Tau constructs were obtained in expression vector pNG2 (a derivative of pET‐3a (Merck‐Novagen, Kenilworth, NJ, USA)). Recombinant proteins were expressed in the *E. coli* BL21 (DE3) strain (Merck‐Novagen) and purified using the heat stability of Tau protein and by FPLC SP‐Sepharose (GE Healthcare Little Chalfont, UK). The cell pellet was resuspended in extraction buffer (50 mm MES, 500 mm NaCl, 1 mm MgSO_4_, 1 mm EGTA, and 5 mm DTT, pH 6.8) with a protease inhibitor mixture (Roche Applied Science, Penzberg, Germany). Cells were disrupted with a French pressure cell, boiled for 20 min and after centrifugation, the supernatant was dialyzed against two changes of cation exchange chromatography buffer A (20 mm MES, 50 mm NaCl, 1 mm MgSO_4_, 1 mm EGTA, 2 mm DTT, and 0.1 mm PMSF, pH 6.8) and loaded on a FPLC SP‐Sepharose column. The protein was eluted with a linear gradient of cation exchange chromatography buffer B (20 mm MES, 1 m NaCl, 1 mm MgSO_4_, 1 mm EGTA, 2 mm DTT, and 0.1 mm PMSF, pH 6.8). The eluate was exchanged to PBS buffer (137 mm NaCl, 3 mm KCl, 10 mm Na_2_HPO_4_, 2 mm KH_2_PO_4_, and 1 mm DTT, pH 7.4) via dialysis. The purity of proteins was ascertained by SDS‐PAGE. Where necessary, breakdown products were removed using the additional gel filtration column Superdex G75 with PBS buffer. In the experimental construct used in our study, all these residues were exchanged for E to generate the pseudophosphorylated protein hTau40 AT8/AT100/PHF‐1 (Zheng‐Fischhofer *et al*., 1998)—in the case of AT8 S^199^, S^202^, and T^205^ (Biernat *et al*., 1992), for AT100 it is T^212^ and S^214^ and for PHF‐1 it is S^396^ and S^404^.

### Measurement of lysosomal activity

*CMA* activity *in vitro* was measured using isolated intact lysosomes incubated with purified proteins at 37 °C in an isosmotic media (20 mm MOPS pH 7.3, 0.25 m sucrose) for 20 min. At the end of the incubation, lysosomes were collected by centrifugation and subjected to immunoblot (Kaushik & Cuervo, 2009). Binding was calculated as the amount of substrate protein bound to the lysosomal membrane in the absence of protease inhibitors and uptake by subtracting the amount of protein associated with lysosomes in the presence (protein bound to the lysosomal membrane and taken up by lysosomes) and absence (protein bound to the lysosomal membrane) of protease inhibitors. Where indicated, lysosomes with lower CMA activity were used in the incubation to determine nonselective binding of the purified proteins to cellular membranes.

*CMA activity in intact cells* was measured using lentivirus‐mediated expression of the KFERQ‐PS‐Dendra2 and high‐content microscopy (Koga *et al*., 2011). Cells were plated in 96‐well plate and photoactivated with a 405‐nm light‐emitting diode (LED; Norlux) for 4 min with the intensity of 3.5 mA (current constant). After 16 h, cells were fixed with 4% paraformaldehyde and images were captured with a high‐content microscope (Operetta system, Perkin Elmer) and quantification was performed with the manufacturer's software in a minimum of 800 cells (approx. nine fields).

### Measurement of endosomal activity

*e‐MI activity in vitro* was measured using isolated late endosomes incubated with purified proteins and subjected to immunoblot (Sahu *et al*., 2011). Binding and internalization were calculated as the amount of substrate protein bound to the late endosomal membrane and intact internal vesicles in the absence of protease inhibitors and luminal degradation by subtracting the amount of protein associated with late endosomes in the presence (protein bound to the endosomal membrane, intact internal vesicles and inside late endosomal lumen) and absence (protein bound to the endosomal membrane and intact internal vesicles) of protease inhibitors.

*e‐MI activity in intact cells* was measured using lentivirus‐mediated expression of the KFERQ‐N‐split Venus and KFERQ‐C‐split Venus and high‐content microscopy (Koga *et al*., 2011). Cells transduced with lentivirus carrying those two parts of split Venus, were plated in 96‐well plates and incubated in complete media supplemented or not with 20 mm NH_4_Cl/100 μm leupeptin (N/L) for 12 h. At the end of the incubation, cells were fixed with 4% paraformaldehyde and images were captured with a high‐content microscope (Operetta system, Perkin Elmer). Quantification was performed with the manufacturer's software in a minimum of 800 cells (approx. nine fields) by measuring number of fluorescent puncta per cell in untreated cells (substrate binding/internalization by e‐MI). The increase in number of puncta upon addition of N/L corresponds to the amount of substrate degraded by e‐MI.

### Measurement of macroautophagy

Macroautophagy was measured in intact cells transduced with the mCherry‐GFP‐LC3 tandem reporter (Klionsky *et al*., 2016). Cells stably transduced with lentivirus carrying this reporter were plated in 96‐well plate and incubated in serum‐supplemented complete media or in serum‐deprived media for 12 h. Where indicated, increasing concentrations of paraquat (PQ) or thapsigargin (TG) were added 6 h after plating. All wells were fixed and imaged in the Operetta system as described in previous sections. The total number of autophagic vacuoles per cell was calculated by quantification of the number of red puncta in cells, the number of autophagosomes as the green puncta per cell, and autophagic flux as the number of only red (autolysosomes) puncta per cell.

### General methods

Protein concentration was determined using the Lowry method with bovine serum albumin as a standard (Lowry *et al*., 1951). Cells were solubilized on ice with RIPA buffer (1% Triton X‐100, 1% sodium deoxycholate, 0.1% SDS, 0.15 m NaCl, 0.01 m sodium phosphate, pH 7.2). Cellular viability was measured in cells plated in 96‐well flat‐bottom plates using the CellTiter Blue cell viability assay reagent (Promega) as changes in the fluorescence (excitation 540 nm, emission 590 nm) according to the manufacturer's instructions. Fluorescence intensity values were normalized to values of untreated wells. Immunoblotting was performed after transferring SDS‐PAGE gels to nitrocellulose membranes (Towbin *et al*., 1979). The proteins of interest were visualized by chemiluminescence using peroxidase‐conjugated secondary antibodies in LAS‐3000 Imaging System (Fujifilm, Tokyo, Japan). Densitometric quantification of the immunoblotted membranes was performed using ImageJ (NIH). When using total cellular protein lysates, actin was used for normalization purposes as contribution of autophagic pathways to its degradation is minimal. A decrease in the relative contribution of actin to the same amount of total cell lysate protein is indicative of accumulation of other proteins.

### Sample size and statistical analysis

For the studies of isolation of lysosomes and cell fractionation, the number of animals for preparation was determined based on the average values of enrichment and recovery of endogenous markers for each compartment. All numerical results are reported as mean + s.e.m., and represent data from a minimum of three independent experiments unless otherwise stated. We determined the statistical significance of the difference between experimental groups in instances of single comparisons by the two‐tailed unpaired Student's *t*‐test with the Sigma Plot software (Jandel Scientific, San Rafael, CA, USA). In instances of multiple means comparisons, we used one‐way analysis of variance followed by the Bonferroni post hoc test to determine statistical significance. Statistical analysis was performed in all the assays, and significant differences are noted in the graphical representations.

## Funding

This work was supported by grants from the National Institutes of Health AG054108, AG031782, NS100717, and AG038072 (AMC) and by the generous support of the Rainwater Charitable Foundation (Tau Consortium) and Robert and Renée Belfer (AMC). EM and EMM acknowledge support from the DZNE, MPG, and the Rainwater Charitable Foundation (Tau Consortium).

## Author contributions

BC performed the studies with purified proteins and cultured cells, prepared the first version of the manuscript, and contributed to the final editing. AD performed the high‐content microscopy and protein turnover studies, IT performed part of the uptake assays, and YRJ performed the viability studies. YW provided all purified proteins and contributed to the editing of the manuscript. EMM, EM, and AMC conceived and directed the study and prepared and edited the final version of the manuscript. BS developed and characterized the eMI reporter. [Correction added on 20 October 2017, after first online publication: The authors' contributions section has been updated in this current version.]

## Conflict of interest

The authors declare no conflict of interest.

## Supporting information


**Fig. S1** Proteolytic susceptibility of different mutant tau proteins.
**Fig. S2** Association of the CMA fluorescent reporter with lysosomes.
**Fig. S3** Resistance to stress of cells expressing disease‐related mutant tau proteins.
**Fig. S4** Effect of disease‐associated mutant tau proteins on macroautophagy flux.
**Fig. S5** Effect of disease‐related mutant tau proteins on macroautophagy activation in response to stress.
**Fig. S6** Summary of the interplay of disease‐related mutant tau proteins with different autophagic pathways.
**Fig. S7** Effect of different mutations and modifications on degradation of tau by CMA (left) or by e‐MI.
