# Supplementary material for: Interplay of pathogenic forms of human tau with different autophagic pathways
Source: Aging Cell. 2017 Oct 12;17(1):e12692. doi: 10.1111/acel.12692 (PMC5770880; doi:10.1111/acel.12692)
Supplement: Supplementary file 1 — Fig. S1 Proteolytic susceptibility of different mutant tau proteins. Fig. S2 Association of the CMA fluorescent reporter with lysosomes. Fig. S3 Resistance to stress of cells expressing disease‐related mutant tau proteins. Fig. S4 Effect of disease‐associated mutant tau proteins on macroautophagy flux. Fig. S5 Effect of disease‐related mutant tau proteins on macroautophagy activation in response to stress. Fig. S6 Summary of the interplay of disease‐related mutant tau proteins with different autophagic pathways. Fig. S7 Effect of different mutations and modifications on degradation of tau by CMA (left) or by e‐MI. [file ACEL-17-na-s001.pdf]

Interplay of Pathogenic Forms of Human Tau with Different Autophagic Pathways

Benjamin Caballero[1,2] Yipeng Wang[3,4], Antonio Diaz[1,2], Inmaculada Tasset[1,2], Yves Robert Juste[1,2], Eva-Maria Mandelkow[3,4], Eckhard Mandelkow[3,4,*] and Ana Maria Cuervo[1,2,*]

SUPPORTING INFORMATION

Supplementary Fig. 1. Proteolytic susceptibility of different mutant tau proteins.

Supplementary Fig. 2. Association of the CMA fluorescent reporter with lysosomes.

Supplementary Fig. 3. Resistance to stress of cells expressing disease-related mutant tau proteins.

Supplementary Fig. 4. Effect of disease-associated mutant tau proteins on macroautophagy flux.

Supplementary Fig. 5. Effect of disease-related mutant tau proteins on macroautophagy activation in response to stress.

Supplementary Fig. 6. Summary of the interplay of disease-related mutant tau proteins with different autophagic pathways.

Supplementary Fig. 7. Effect of different mutations and modifications on degradation of tau by CMA (left) or by e-MI

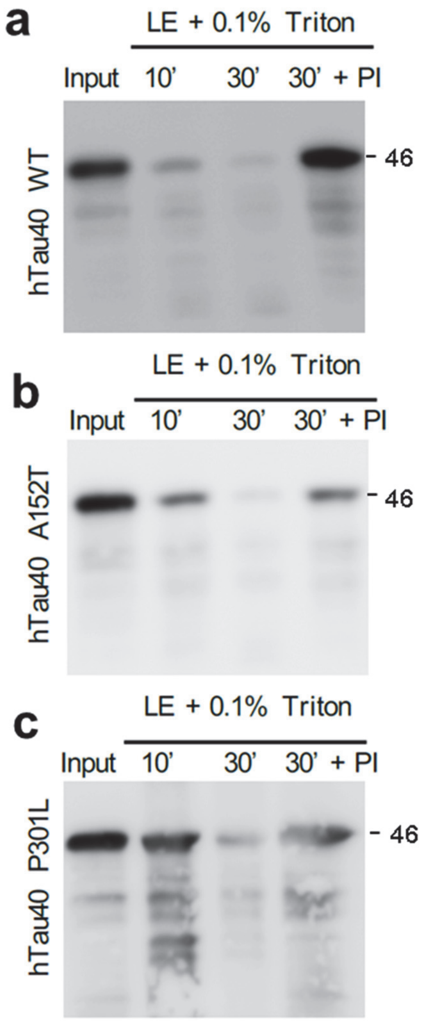

Supplementary Fig. 1. Proteolytic susceptibility of different mutant tau proteins. Immunoblots for tau of late endosomes (LE) pre-incubated with 0.1% Triton X-100 for 10 min on ice to disrupt the LE membrane and then incubated with 0.5 µg of recombinant wild-type (WT) (**a**), A152T (**b**) or P301L (**c**) mutant tau (full-length, 2N4R) for the indicated times at 37ºC. Input: 1/10 of tau added to the incubation. Where indicated, a cocktail of protease inhibitors (PI) was added into the incubation media.

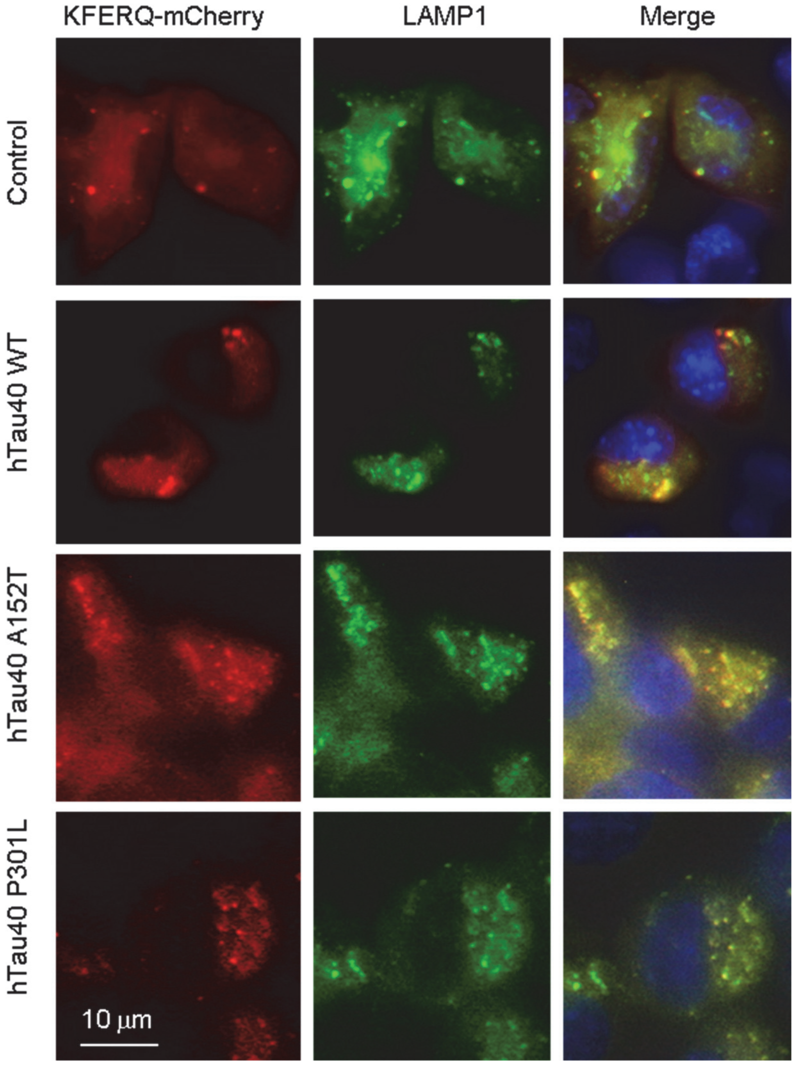

Supplementary Fig. 2. Association of the CMA fluorescent reporter with lysosomes. Representative images of N2a cells expressing the indicated tau proteins and transduced with the lentivirus carrying the KFERQ-PA-mCherry reporter and immunostained with an antibody against LAMP-1 to highlight endo/lysosomal compartments. Note that in conditions with low CMA activity all KFERQ-PA-mCherry positive puncta is positive for LAMP-1, but that, as expected, not all LAMP1 positive compartments are positive for the CMA reporter since not all lysosomes are active for CMA. Nuclei were stained with DAPI.

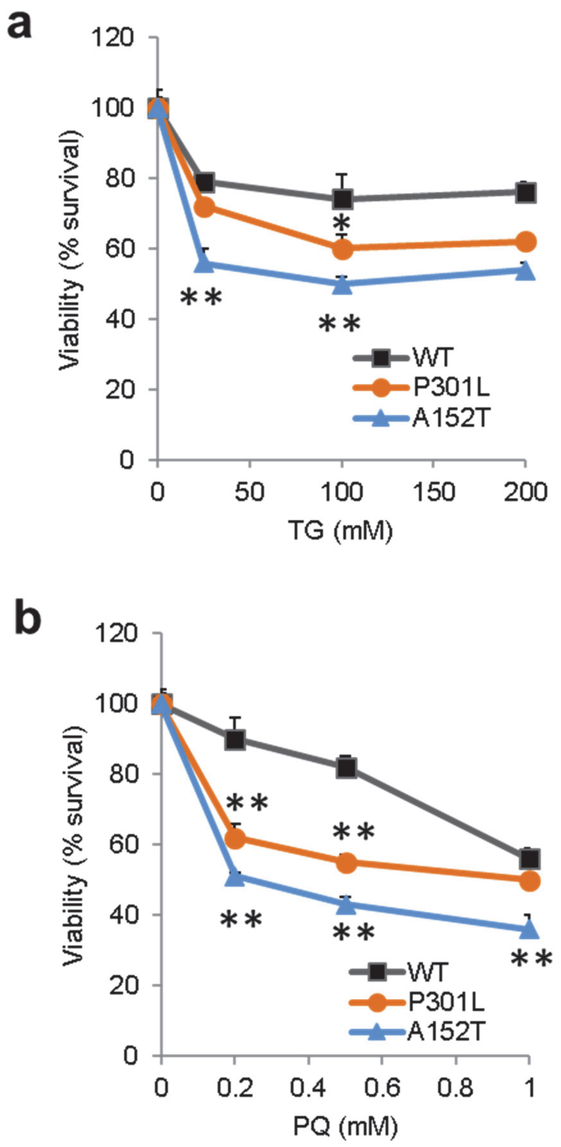

Supplementary Fig. 3. Resistance to stress of cells expressing disease-related mutant tau proteins. Mouse neuroblastoma cell lines Neuro-2a (N2a) treated with doxycycline to activate expression of the indicated tau proteins were incubated with the indicated concentrations of thapsigargin (TG) (**a**) or paraquat (PQ) (**b**) and cellular viability was measured after 12 h. Viability values were calculated as the percentage of cells alive relative to the number of cells in untreated wells. Values are mean±s.e.m. of three independent wells per concentration. Differences with cells expressing WT tau were significant for *p<0.05 and ** p<0.01.

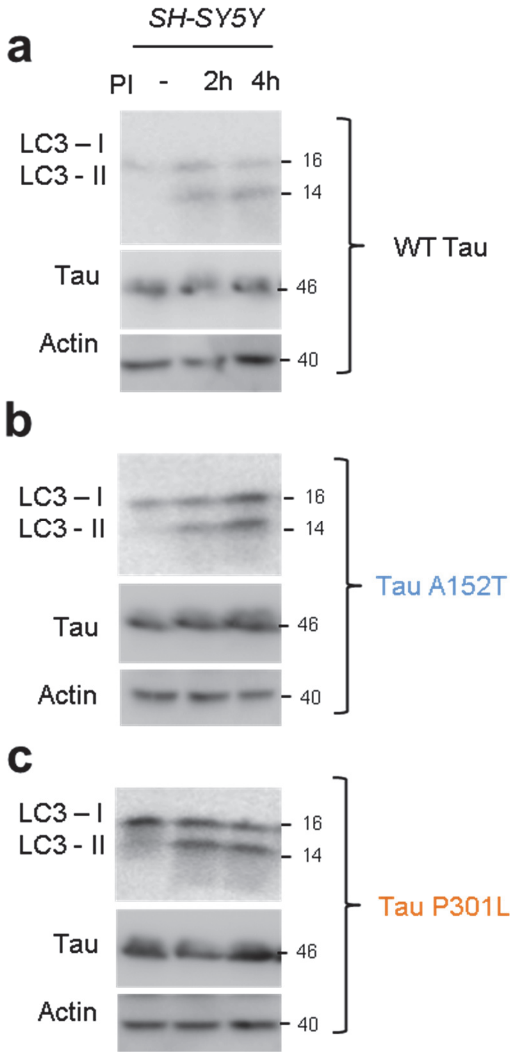

Supplementary Fig. 4. Effect of disease-associated mutant tau proteins on macroautophagy flux. Immunoblots for LC3 of neuroblastoma SH-SY5Y cells transiently transfected with plasmids expressing full length (2N4R) wild-type (WT) (**a**), A152T (**b**) or P301L (**c**) tau and incubated without (-) or with NH_4Cl 20mM and leupeptin 200μM for 2 or 4 hours. Actin is shown as loading control and tau as a control for protein expression (note that the short times of incubation with the inhibitors does not allow for significant accumulation of tau proteins because they have a considerably longer high-life than LC3).

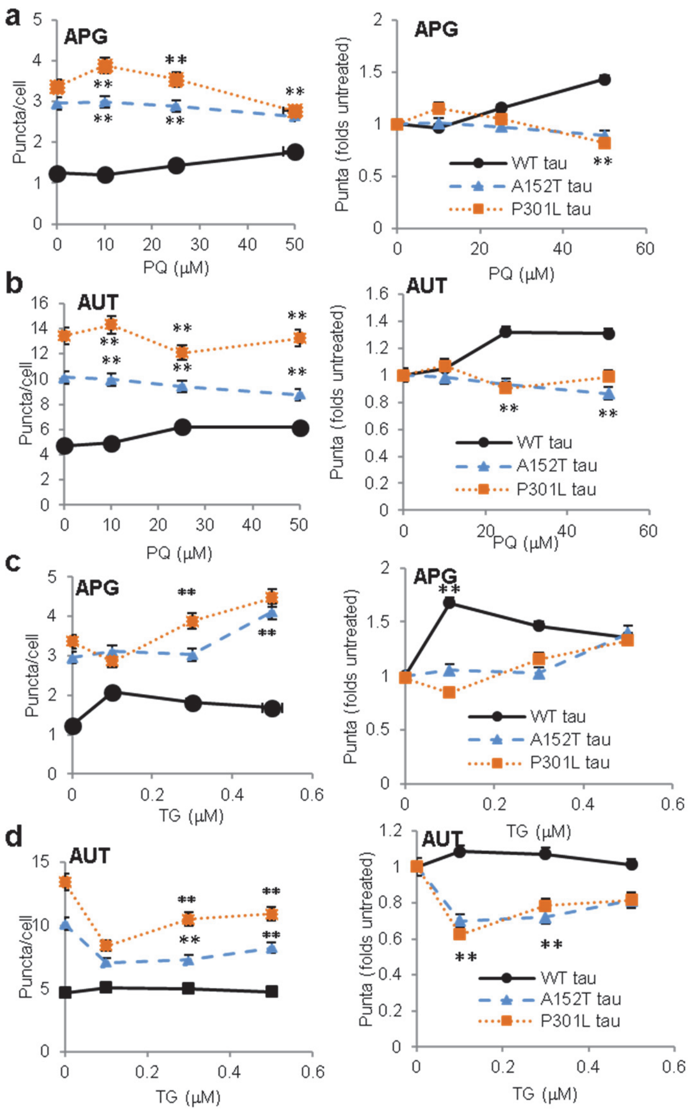

Supplementary Figure 5. Effect of disease-related mutant tau proteins on macroautophagy activation in response to stress. Mouse neuroblastoma cell lines Neuro-2a (N2a) treated with doxycycline to activate expression of the indicated full length (2N4R) tau proteins and transduced with the mCherry-GFP-LC3 reporter were maintained in presence of increasing concentrations of paraquat (PQ, **a**,**b**) or thapsigargin (TG, **c**,**d**). Quantification of total number (left) or number relative to untreated cells (right) of autophagosomes (APG, yellow puncta, **a**,**c**) and autolysosomes (AUT, red only puncta, **b**,**d**). n> 800 cells/condition in three experiments with triplicate wells. All values are mean±s.e.m. Differences with cells expressing WT tau were significant for *p<0.05 and **p<0.01.

a Tau degradation

	WT	A152T	P301L
CMA	X	X	x
eMI	x		
MA		X	

b Effect of Tau on Autophagy

	serum+		
	WT	A152T	P301L
CMA			UP
eMI	down	down	down
MA		UP	UP
proteo		UP	

	serum-		
	WT	A152T	P301L
CMA		UP	
MA		UP	UP
proteo		UP	UP

Supplementary Figure 6. Summary of the interplay of disease-related mutant tau proteins with different autophagic pathways. Schematic model summarizing the main differences on the degradation by autophagy (**a**) of the two different disease-related mutant tau proteins compared in this study and of their effect on basal (serum+) and inducible (serum-) activities of different autophagic pathways and proteasome (proteo) (**b**). The intensity of blue in **a** indicates rates of degradation. Grey: no degradation. In **b**, red indicates inhibition, green activation and grey no significant change.

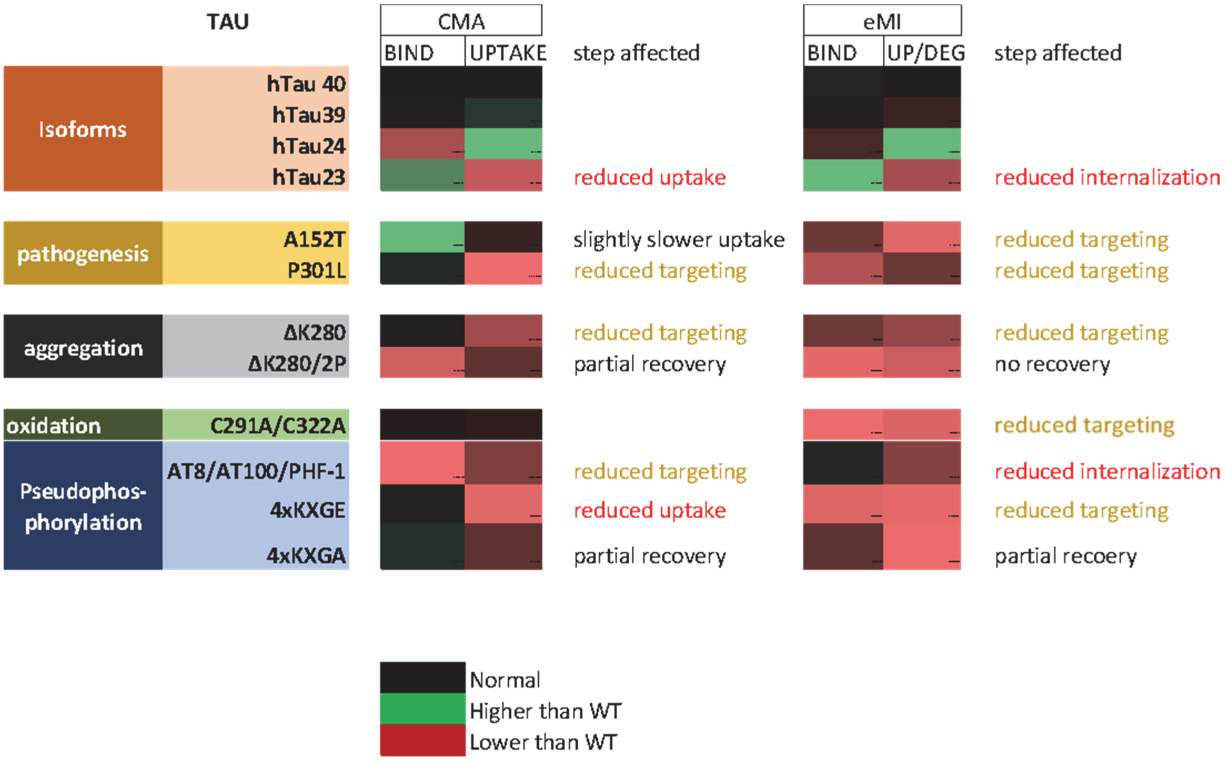

Supplementary Figure 7. Effect of different mutations and modifications on degradation of tau by CMA (left) or by eMI (right). Heatmap of the quantitative differences in binding (BIND) and uptake by CMA-active lysosomes (right) or binding or uptake plus degradation (UP/DEG) by late endosomes (right) of the indicated tau proteins relative to the values of hTau40 that was assigned an arbitrary value of 1. Values above 1 are in green and values below 1 in red. The affected CMA and eMI steps and the effect of proline replacement (in the case of ΔK280 tau) or replacement of E by A (in the pseudophosphorylation mutants) are indicated on the right.
